# Genetic variants associated with susceptibility to idiopathic pulmonary fibrosis in people of European ancestry: a genome-wide association study

**DOI:** 10.1016/S2213-2600(17)30387-9

**Published:** 2017-11

**Authors:** Richard J Allen, Joanne Porte, Rebecca Braybrooke, Carlos Flores, Tasha E Fingerlin, Justin M Oldham, Beatriz Guillen-Guio, Shwu-Fan Ma, Tsukasa Okamoto, Alison E John, Ma'en Obeidat, Ivana V Yang, Amanda Henry, Richard B Hubbard, Vidya Navaratnam, Gauri Saini, Norma Thompson, Helen L Booth, Simon P Hart, Mike R Hill, Nik Hirani, Toby M Maher, Robin J McAnulty, Ann B Millar, Philip L Molyneaux, Helen Parfrey, Doris M Rassl, Moira K B Whyte, William A Fahy, Richard P Marshall, Eunice Oballa, Yohan Bossé, David C Nickle, Don D Sin, Wim Timens, Nick Shrine, Ian Sayers, Ian P Hall, Imre Noth, David A Schwartz, Martin D Tobin, Louise V Wain, R Gisli Jenkins

**Affiliations:** a Department of Health Sciences, University of Leicester, Leicester, UK; b Division of Respiratory Medicine, University of Nottingham, Nottingham, UK; c National Institute for Health Research, Nottingham Biomedical Research Centre, Nottingham University Hospitals, Nottingham, UK; d Nottingham Molecular Pathology Node, University of Nottingham, Nottingham, UK; e Division of Epidemiology and Public Health, University of Nottingham, Nottingham, UK; f Research Unit, Hospital Universitario NS de Candelaria, Universidad de La Laguna, Santa Cruz de Tenerife, Spain; g CIBER de Enfermedades Respiratorias, Instituto de Salud Carlos III, Spain; h Instituto Tecnológico y de Energías Renovables (ITER, S.A.), Santa Cruz de Tenerife, Spain; i Center for Genes, Environment and Health, National Jewish Health, Denver, CO, USA; j Department of Biostatistics and Informatics, University of Colorado, Denver, CO, USA; k Department of Internal Medicine, University of California Davis, Davis, CA, USA; l Section of Pulmonary and Critical Care Medicine, University of Chicago, Chicago, IL, USA; m Department of Medicine, University of Colorado Denver, Denver, CO, USA; n The University of British Columbia Centre for Heart Lung Innovation, St Paul's Hospital, Vancouver, BC, Canada; o Department of Thoracic Medicine, University College London Hospitals, London, UK; p Respiratory Research Group, Centre for Cardiovascular and Metabolic Research, The Hull York Medical School, Hull, UK; q Clinical Trial Service Unit & Epidemiological Studies Unit, Nuffield Department of Population Health, University of Oxford, Oxford, UK; r MRC Centre for Inflammation Research at the University of Edinburgh, Edinburgh, UK; s NIHR Respiratory Biomedical Research Unit, Royal Brompton Hospital, London, UK; t Fibrosis Research Group, Inflammation, Repair and Development Section, National Heart and Lung Institute, Imperial College, London, UK; u UCL Respiratory Centre for Inflammation and Tissue Repair, University College London, London, UK; v Academic Respiratory Unit, School of Clinical Sciences, University of Bristol, Bristol, UK; w Respiratory Medicine, Papworth Hospital, Cambridge, UK; x Department of Pathology, Papworth Hospital, Cambridge, UK; y Fibrosis Discovery Performance Unit, GlaxoSmithKline, Stevenage, UK; z Institut Universitaire de Cardiologie et de Pneumologie de Québec, Department of Molecular Medicine, Laval University, Quebec City, QC, Canada; aa Merck Research Laboratories, Genetics and Pharmacogenomics, Boston, MA, USA; ab Respiratory Division, Department of Medicine, University of British Columbia, Vancouver, BC, Canada; ac Department of Pathology and Medical Biology, University Medical Centre Groningen, Groningen Research Institute for Asthma and COPD, University of Groningen, Groningen, Netherlands; ad Department of Immunology, University of Colorado Denver, Denver, CO, USA; ae National Institute for Health Research, Leicester Respiratory Biomedical Research Centre, Glenfield Hospital, Leicester, UK

## Abstract

**Background:**

Idiopathic pulmonary fibrosis (IPF) is a chronic progressive lung disease with high mortality, uncertain cause, and few treatment options. Studies have identified a significant genetic risk associated with the development of IPF; however, mechanisms by which genetic risk factors promote IPF remain unclear. We aimed to identify genetic variants associated with IPF susceptibility and provide mechanistic insight using gene and protein expression analyses.

**Methods:**

We used a two-stage approach: a genome-wide association study in patients with IPF of European ancestry recruited from nine different centres in the UK and controls selected from UK Biobank (stage 1) matched for age, sex, and smoking status; and a follow-up of associated genetic variants in independent datasets of patients with IPF and controls from two independent US samples from the Chicago consortium and the Colorado consortium (stage 2). We investigated the effect of novel signals on gene expression in large transcriptomic and genomic data resources, and examined expression using lung tissue samples from patients with IPF and controls.

**Findings:**

602 patients with IPF and 3366 controls were selected for stage 1. For stage 2, 2158 patients with IPF and 5195 controls were selected. We identified a novel genome-wide significant signal of association with IPF susceptibility near A-kinase anchoring protein 13 (*AKAP13*; rs62025270, odds ratio [OR] 1·27 [95% CI 1·18–1·37], p=1·32 × 10^−9^) and confirmed previously reported signals, including in mucin 5B (*MUC5B*; rs35705950, OR 2·89 [2·56–3·26], p=1·12 × 10^−66^) and desmoplakin (*DSP*; rs2076295, OR 1·44 [1·35–1·54], p=7·81 × 10^−28^). For rs62025270, the allele A associated with increased susceptibility to IPF was also associated with increased expression of *AKAP13* mRNA in lung tissue from patients who had lung resection procedures (n=1111). We showed that *AKAP13* is expressed in the alveolar epithelium and lymphoid follicles from patients with IPF, and *AKAP13* mRNA expression was 1·42-times higher in lung tissue from patients with IPF (n=46) than that in lung tissue from controls (n=51).

**Interpretation:**

*AKAP13* is a Rho guanine nucleotide exchange factor regulating activation of RhoA, which is known to be involved in profibrotic signalling pathways. The identification of *AKAP13* as a susceptibility gene for IPF increases the prospect of successfully targeting RhoA pathway inhibitors in patients with IPF.

**Funding:**

UK Medical Research Council, National Heart, Lung, and Blood Institute of the US National Institutes of Health, Agencia Canaria de Investigación, Innovación y Sociedad de la Información, Spain, UK National Institute for Health Research, and the British Lung Foundation.

## Introduction

Idiopathic pulmonary fibrosis (IPF) is a chronic, progressive, fibrotic lung disease of unknown cause with a poor prognosis. The incidence of IPF in the UK is about 4·5–7·5 per 100 000 and is increasing,^1^ with a median survival of approximately 3 years.^2^, ^3^ Since 2010, two new therapies, pirfenidone and nintedanib, have been approved for the treatment of IPF, but these treatments only slow disease progression and do not halt or reverse pulmonary fibrosis.^4^, ^5^ Furthermore, these drugs are not universally effective and the mechanisms of their antifibrotic effects are unknown. Therefore, a detailed understanding of the genetic risk factors for IPF and their associated molecular pathways is urgently required.

The current theory suggests that IPF is characterised by initial damage to the alveolar epithelium, which then signals to various cell types, predominantly fibroblasts and macrophages, promoting tissue damage and extracellular matrix synthesis that leads to parenchymal destruction and alveolar replacement by dense fibrotic tissue.^2^ Studies from patients with familial pulmonary fibrosis have identified telomerase-related and surfactant protein-related genes that are associated with epithelial dysfunction.^6^, ^7^, ^8^ Similarly, genome-wide association studies (GWAS) of IPF have reported various independent genetic association signals related to epithelial cell function of genome-wide significance,^9^, ^10^, ^11^, ^12^ including lung defence (such as mucin 5B [*MUC5B*]), telomere maintenance (such as telomerase reverse transcriptase [*TERT*] and CST complex subunit [*STN1*]) and cell–cell adhesion (such as desmoplakin [*DSP*] and dipeptidyl peptidase 9 [*DPP9*]).^13^ Telomerase mutations are associated with short telomeres,^8^ which lead to increased disease progression,^14^ whereas *MUC5B* and Toll interacting protein (*TOLLIP*) variants are associated with reduced disease progression.^10^, ^15^ However, these genetic abnormalities account for only about 30% of the genetic risk associated with IPF, and the molecular mechanisms that become dysregulated remain to be established.^13^

**Research in context**

**Evidence before this study**

We searched Web of Science between Aug 21, 2015, and June 7, 2017, with the search terms “pulmonary fibrosis” and “genome wide” with no restrictions on publication date or language. Previous genome-wide association studies (GWAS) have reported 16 independent signals associated, at genome-wide significance, with susceptibility to idiopathic pulmonary fibrosis (IPF). Although previous studies have shown that epithelial cell function, lung defence, cell–cell adhesion, and telomere maintenance might play important roles in IPF, the precise mechanisms through which the genes identified in previous GWAS promote IPF are still poorly understood.

**Added value of this study**

To our knowledge, this study is the largest genetic study of IPF done to date, bringing together 2760 patients with IPF and 8561 controls. We present a novel genome-wide significant genetic association signal and novel gene expression data that implicate A-kinase anchoring protein 13 (*AKAP13*) as an IPF susceptibility gene. AKAP13 is a Rho guanine nucleotide exchange factor (RhoGEF) that regulates activation of Rho A. RhoA is a molecule with a known role in profibrotic signalling pathways; however, AKAP13 has not previously been implicated in the pathogenesis of IPF. These studies provide genetic veracity for targeting Rho signalling in IPF.

**Implications of all the available evidence**

The identification of an association between AKAP13, an epithelial RhoGEF, and IPF supports the role of epithelial processes in IPF pathogenesis. Furthermore, AKAP13 has a role in a pharmacologically tractable molecular pathway that could lead to new treatments for IPF.

Identifying further genetic risk factors will enable the identification of molecular pathways involved in the pathogenesis of IPF that could potentially be targeted with novel treatments. Molecular targets with supporting genetic evidence are twice as likely to be successful in clinical development as those with no genetic support.^16^ We designed and implemented the largest UK-based IPF case-control GWAS to date (stage 1) and followed up promising genetic association signals in two independent IPF case-control studies (stage 2). We aimed to identify genetic variants associated with IPF susceptibility and provide mechanistic insight into those genetic association signals using gene and protein expression analyses.

## Methods

### Study design

This GWAS used a two-stage approach to identify novel genome-wide significant signals associated with susceptibility to IPF. In stage 1, a GWAS was done using patients with IPF from nine different centres in the UK and controls from UK Biobank. Variants showing a significant association (p<5 × 10^−6^) in stage 1 were then further analysed in independent samples of patients with IPF and controls from two US samples, the Colorado consortium and the Chicago consortium (stage 2). We defined statistically significant associations with susceptibility to IPF as variants that met genome-wide significance (p<5 × 10^−8^) after meta-analysis of stages 1 and 2 together. Variants that became less significant in the meta-analysis than in stage 1 alone were not reported as showing an association.

Stage 1 comprised patients with IPF selected from nine different centres across the UK (appendix p 1). All diagnoses were made in accordance with accepted international criteria.^17^, ^18^, ^19^ Controls were selected from UK Biobank and were matched for age, sex, and smoking distributions, showed no evidence of having any interstitial lung disease, and had genetic data available (appendix p 1). Stringent quality control testing of all samples was done, such as removing individuals with poor call rates, heterozygosity outliers, duplicates, related individuals, ancestry outliers, and sex mismatches (appendix p 1). All individuals were of European ancestry.

Stage 2 comprised two additional independent case-control studies that have been previously described by Noth and colleagues^10^ (Chicago consortium) and Fingerlin and colleagues^11^ (Colorado consortium). For both these studies, patients with IPF were diagnosed using American Thoracic Society and European Respiratory Society guidelines.^17^, ^18^ All studies had appropriate institutional review board or ethics approval.

### Procedures and statistical analysis

For stage 1, patients with IPF and a third of the UK Biobank controls were genotyped using the Affymetrix Axiom UK BiLEVE array (Affymetrix, Santa Clara, CA, USA). The remainder of the controls were genotyped using the Affymetrix Axiom UK Biobank array (95% identical to the UK BiLEVE array). Genotyping and imputation procedures for studies contributing to stage 1 and 2 are described in the appendix p 1. Because the UK Biobank controls genotyped on the Axiom UK BiLEVE array had originally been selected on the basis of lung function and smoking behaviour, it was reasonable to assume that allele frequency differences between the controls genotyped on the two arrays could feasibly be driven by either technical array artefacts or genuine associations with lung function and smoking. To account for this in our analysis, and to avoid incorrectly reporting associations driven by array as associations with IPF susceptibility, we did additional sensitivity analyses (appendix p 2).

The genome-wide association analysis of IPF susceptibility was done assuming an additive genetic effect and conditioning on age, sex, and the first ten principal components to adjust for ancestry.^20^ The analysis was run using a score test because of its computational efficiency, using SNPTEST^21^ version 2.5.2. For variants with minor allele count less than 400 and score test p<5 × 10^−3^, the analysis was rerun using the Firth test using EPACTS^22^ version 3.2.4 (appendix p 2).

Independent variants reaching a threshold of p<5 × 10^−6^ in association testing in stage 1 were followed up in stage 2. Conditional analyses were used to identify additional independent signals in the same genomic region (appendix p 2).

In the Chicago consortium, analyses were done using the Firth test, adjusting for age and sex. In the Colorado consortium, analyses were done using the Firth test on a logistic regression model, adjusting for sex and the top three dimensions from a multidimensional scaling model.

For variants that were significantly associated with IPF susceptibility in stage 1 only after conditioning on another variant, the analysis was run in stage 2 conditioning on the same variant. The results from these stage 2 studies were meta-analysed using a fixed-effects model.

For variants that showed a significant (p<5 × 10^−6^) association with IPF risk in stage 1, available association test statistics from stages 1 and 2 were meta-analysed using a fixed-effects model. Signals that had genome-wide significance (p<5 × 10^−8^) when meta-analysing stages 1 and 2 were reported as significantly associated with susceptibility to IPF. A Bayesian approach was used to fine-map those signals to create 95% credible sets (a set of variants that was 95% likely to contain the causal variant; appendix p 3).

To investigate whether the variants showing an association with susceptibility to IPF were also associated with survival time in IPF, a Cox proportional hazards model was fitted in a subset of the stage 1 patients with IPF that had data for survival time. The model made adjustments for age, sex, the first ten principal components, and the recruiting study centre. Analysis was done using the Survival package in R version 2.2.3.

We searched for evidence that genetic variants associated with susceptibility to IPF were independently associated with expression of a particular gene, because an association could implicate a particular gene as the driver of the signal. Variants that were significantly associated with susceptibility to IPF and proxy variants (correlated with linkage disequilibrium *r*^2^>0·8) were investigated in three expression quantitative trait locus (eQTL) datasets: a lung eQTL database comprising individuals from three cohorts (University of British Columbia, Laval, and Groningen), a blood eQTL database, and in the Genotype-Tissue Expression project (GTEx) cohort (multiple tissues; appendix p 3).

RT-PCR gene expression analysis was done on RNA extracted from human lung tissues of 46 patients with IPF and 51 controls from the Lung Tissue Research Consortium using standard methods (appendix p 3). Initially, differential gene expression in the lung was compared between the patients with IPF and controls using two-tailed Student's *t* test comparing the change in C_T_ values between groups. Linear regression analysis was used to compare A-kinase anchoring protein 13 (*AKAP13*) expression in controls and patients with IPF while also controlling for age, smoking (ever or never and pack-years), and sex.

Formalin-fixed paraffin-embedded human lung tissue sections were obtained from tissue distant from the tumour that was obtained during lung resection from controls and tissue from patients with IPF taken at either post-mortem examination or lung transplantation. Immunohistochemistry was done using standard methods (appendix p 3).

We performed in-silico analyses to establish whether known drugs target the proteins identified in the genome-wide and expression analyses or the proteins that interact with them (appendix p 4).

### Role of the funding source

The funders had no role in the study design. GlaxoSmithKline Research and Design participated in collection of data and had access to the raw data from a subset of the UK IPF patient data. LVW and RGJ were involved in all stages of study development and delivery, had full access to all data in the study, and had final responsibility for the decision to submit for publication.

## Results

**Figure 1:**
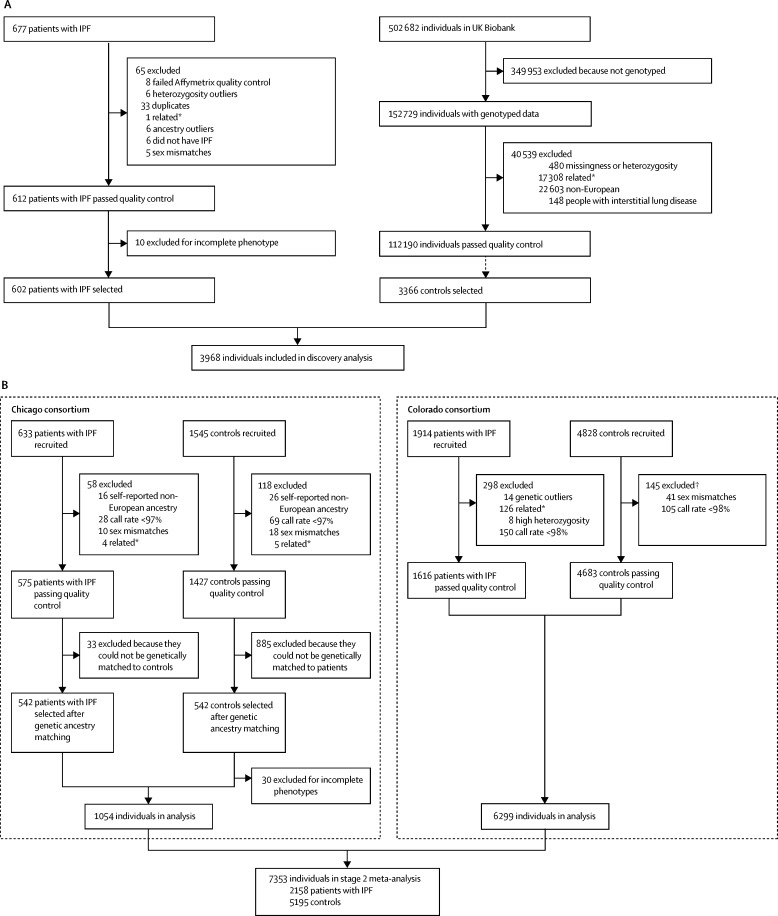
Quality control and sample selection flow chart (A) Stage 1 genome-wide association study. (B) Stage 2 follow-up analyses. *On identification of a pair of individuals who were second-degree relatives or closer, one individual was excluded. IPF=idiopathic pulmonary fibrosis. †One patient had both call rate <98% and sex mismatch.

**Figure 2:**
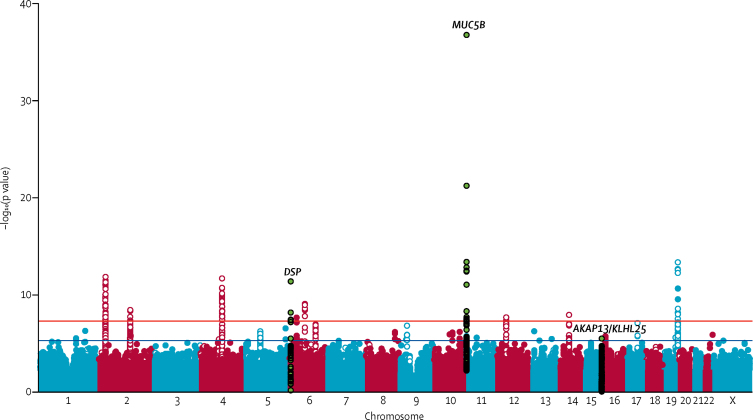
Manhattan plot for the discovery genome-wide association study of IPF susceptibility The x axis shows chromosomal position and the y axis shows –log_10_(p value) from the discovery (stage 1) case-control analysis. Green variants are those that reached genome-wide significance in the meta-analysis of stage 1 and 2 results (and any variant in linkage disequilibrium with the lead variant [*r*^2^>0·1]). The blue line shows the threshold used for selecting variants for stage 2 (p=5 × 10^−6^) and the red line shows genome-wide significance (p=5 × 10^−8^). Hollow circles show variants showing an association with genotyping array in the controls, and those that did not show an association with IPF in stage 2 (appendix pp 2 and 6). IPF=idiopathic pulmonary fibrosis.

After sample quality control testing and genotype imputation, 602 patients with IPF and 3366 UK Biobank controls (selected on April 18, 2016) were included in the stage 1 analysis of 13 076 821 variants (figure 1A). The patients were diagnosed between June, 1996, and July, 2013, with the exception of 52 patients who were historical cases with unknown dates of diagnosis. In total, 44 independent signals were associated with susceptibility to IPF (p<5 × 10^−6^) and were followed up in stage 2 (figure 2).

**Figure 3:**
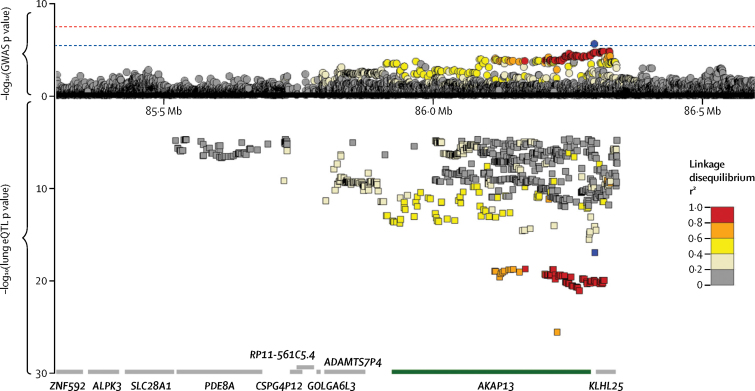
Comparison of case-control association results and lung eQTL results Region plots for a 2 Mb region on chromosome 15 for the stage 1 case-control GWAS (circles above the x axis) and lung eQTL analysis (squares below the x axis). The x axis shows chromosomal position. The y axis above the x-axis shows the –log_10_(p value) from the case-control analysis and the y axis below the x axis shows the –log_10_(p value) for *AKAP13* expression from the lung eQTL analysis. The blue dotted line shows the significance threshold (p=5 × 10^−6^) used in stage 1 and the red dotted line shows genome-wide significance (p=5 × 10^−8^). Boxes at the bottom show gene locations plotted against the same x axis as the case-control and eQTL results, with *AKAP13* highlighted in green. Variants are coloured according to linkage disequilibrium with rs62025270 (shown in blue). eQTL=expression quantitative trait locus. GWAS=genome-wide association study.

A total of 2158 patients with IPF and 5195 controls were included in stage 2 (figure 1B, table 1). Of the 44 variants identified in stage 1, data were available for 27 variants in one or both of the stage 2 studies (appendix pp 8–9). Of these 27 variants, three were significant (p<1·85 × 10^−3^, after Bonferroni correction for 27 tests) in stage 2, and had genome-wide significance (p<5 × 10^−8^) in the meta-analysis of stage 1 and 2 together (table 2). A novel association for the single-nucleotide polymorphism (SNP) rs62025270, which was the most strongly associated SNP in a broad signal covering *AKAP13* and kelch like family member 25 (*KLHL25*; figure 3), had genome-wide significance in the meta-analysis of stages 1 and 2 (minor allele A; odds ratio [OR] 1·27 [95% CI 1·18–1·37], p=1·32 × 10^−9^). This SNP was also significant in stage 2 alone after Bonferroni adjustment for 27 tests (OR 1·22 [95% CI 1·11–1·33], p=9·96 × 10^−6^). The 95% credible set (a set of variants that was 95% likely to contain the causal variant) produced for this signal contained 113 variants (appendix pp 10–11). The other two genome-wide significant signals were previously reported variants in *MUC5B* (rs35705950) and *DSP* (rs2076295).^10^, ^11^, ^12^

**Table 1:** Baseline characteristics of stage 1 and stage 2 samples

	**Stage 1**		**Stage 2: Chicago consortium**		**Stage 2: Colorado consortium**	
	IPF (n=602)	Controls (n=3366)	IPF (n=542)	Controls (n=512)	IPF (n=1616)	Controls (n=4683)
**Age, years**						
Mean (SD)	70 (8·4)	65 (5·5)	68 (3·0)	63* (7·5)	66 (9·5)	..
**Sex**						
Men	426 (71%)	2356 (70%)	385 (71%)	242 (47%)	1091 (68%)	2290 (49%)
Women	176 (29%)	1010 (30%)	157 (29%)	270 (53%)	525 (33%)	2393 (51%)

Data are n (%) unless otherwise stated. Age was not available for Colorado consortium controls. IPF=idiopathic pulmonary fibrosis.

* Age data were only available for 103 individuals.

**Table 2:** Gene variants with genome-wide significance for idiopathic pulmonary fibrosis

	**Chr**	**Position**	**Locus**	**Minor allele**	**Major allele**	**MAF**	**Stage 1**		**Stage 2**		**Meta-analysis (stages 1+2)**	
							OR (95% CI)	p value	OR (95% CI)	p value	OR (95% CI)	p value
rs2076295	6	7563232	*DSP*	G	T	46·3%	1·67 (1·44–1·92)	4·14 × 10^−12^	1·39 (1·29–1·50)	2·47 × 10^−18^	1·44 (1·35–1·54)	7·81 × 10^−28^
rs35705950	11	1241221	*MUC5B*	T	G	14·3%	4·11 (3·31–5·11)	1·86 × 10^−37^	2·46 (2·13–2·85)	3·13 × 10^−34^	2·89 (2·56–3·26)	1·12 × 10^−66^
rs62025270	15	86300198	*AKAP13/KLHL25*	A	G	24·7%	1·49 (1·26–1·76)	3·11 × 10^−6^	1·22 (1·11–1·33)	9·96 × 10^−6^	1·27 (1·18–1·37)	1·32 × 10^−9^

Results from case-control analyses for the variants that were significant (after correction for multiple testing) in stage 2 and reached genome-wide significance in the meta-analysis of stages 1 and 2. MAF corresponds to that from the stage 1 study. ORs were calculated using the minor allele as the effect allele. Stage 2 ORs and p values correspond to the meta-analysis of the Chicago and Colorado consortia results. Chr=chromosome. MAF=minor allele frequency. OR=odds ratio.

We explored whether genotyping array differences could have influenced the association test result for SNP rs62025270. In the control–control comparison (appendix p 6), rs62025270 showed no association with the array (p=0·35, appendix p 12). We repeated the association analysis for rs62025270 restricted to controls who were genotyped on the same array as the patients (602 patients *vs* 1231 controls); the results were consistent with the original analysis (stage 1 OR 1·45, [95% CI 1·20–1·74], p=1·19 × 10^−4^; meta-analysis OR 1·25 [95% CI 1·17–1·33], p=1·86 × 10^−8^). We reimputed the region with only variants genotyped on both arrays using the new Haplotype Reference Consortium imputation reference panel;^23^ the results were again consistent with the original analysis (stage 1 OR 1·48 [95% CI 1·25–1·75], p=4·24 × 10^−6^; meta-analysis OR 1·27 [95% CI 1·19–1·35], p=1·55 × 10^−9^).

Previous GWAS^9^, ^10^, ^11^, ^12^ reported 16 independent signals associated with susceptibility to IPF with genome-wide significance. In stage 1 of our study, which analysed previously unreported data only, nine of these signals had a p value less than 0·05 and a direction of effect consistent with the previously reported effects. Furthermore, seven signals met a Bonferroni adjusted significance threshold for 16 tests (p<3·13 × 10^−3^) with a consistent direction of effect to previous GWAS; namely variants in *MUC5B, DSP*, family with sequence similarity 13 member A (*FAM13A*), *TERT*, mucin 2 (*MUC2*), *DPP9*, and *TOLLIP* (appendix p 13). Five of the remaining seven signals that were not significant had a consistent direction of effect with previous GWAS.

**Figure 4:**
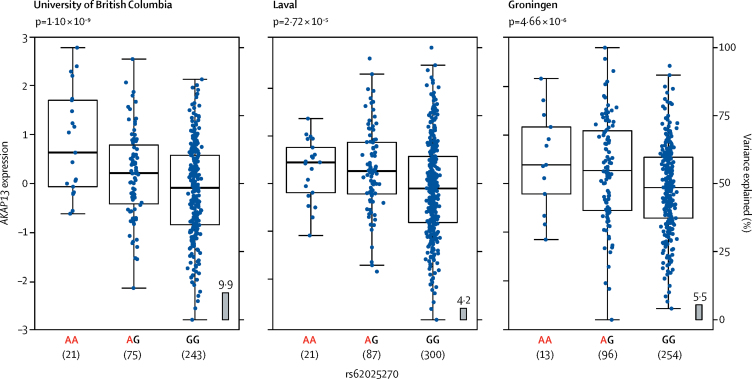
Lung eQTL results for *AKAP13* expression by rs62025270 genotype Three independent datasets are shown. Blue points show normalised residuals of expression of *AKAP13* after adjusting for age, sex, and smoking status for each individual by genotype of rs62025270. The box and whiskers show the mean and IQR for each genotype (left-hand y-axis). The grey boxes show the percentage of variance of *AKAP13* expression that is explained by rs62025270 (right-hand y-axis). The p value relating the genotype to expression is shown at the top for each sample. Red allele indicates the allele associated with increased IPF susceptibility. eQTL=expression quantitative trait locus.

SNP rs62025270[A] was associated with increased expression of *AKAP13* in the lung. To identify the functional consequence of the rs62025270 polymorphism, we assessed associations between rs62025270 and nearby variants and expression of genes (eQTLs). In non-diseased whole-lung tissue from the lung eQTL databases (University of British Columbia n=339, Laval n=409, and Groningen n=363), the minor allele A of rs62025270, associated with increased susceptibility to IPF, was associated with increased expression of *AKAP13* (figure 4). SNP rs62025270 is in linkage disequilibrium (*r*^2^=0·60) with rs17636666, the variant that was most significantly associated with expression of *AKAP13* in lung tissue. This co-localisation of IPF susceptibility association signal and strongest *AKAP13* gene expression association signal (eQTL) in lung tissue (figure 3) further suggests that altered expression of *AKAP13* has a role in IPF susceptibility. The minor allele A of rs62025270 (via proxy SNP rs2554, r^2^=0·93) was also associated with expression of *AKAP13* in whole blood (n=5311; appendix pp 7 and 14), although it was not in linkage disequilibrium (*r*^2^=0·03) with the SNP most strongly associated with *AKAP13* expression in blood (rs870689). This finding suggests differences in regulation of *AKAP13* expression between lung and whole blood, which have different cell-type compositions. Furthermore, the minor allele A of rs62025270 was associated with decreased expression of *AKAP13* in blood, rather than increased expression as seen in lung tissue. The SNP rs62025270 was also found to be associated with expression of RNA genes RP11–158M2.3, RP11–158M2.4, RP11–158M2.5, RP11–815J21.3, and RP11–815J21.4 in a range of tissues in GTEx (appendix p 14).

**Figure 5:**
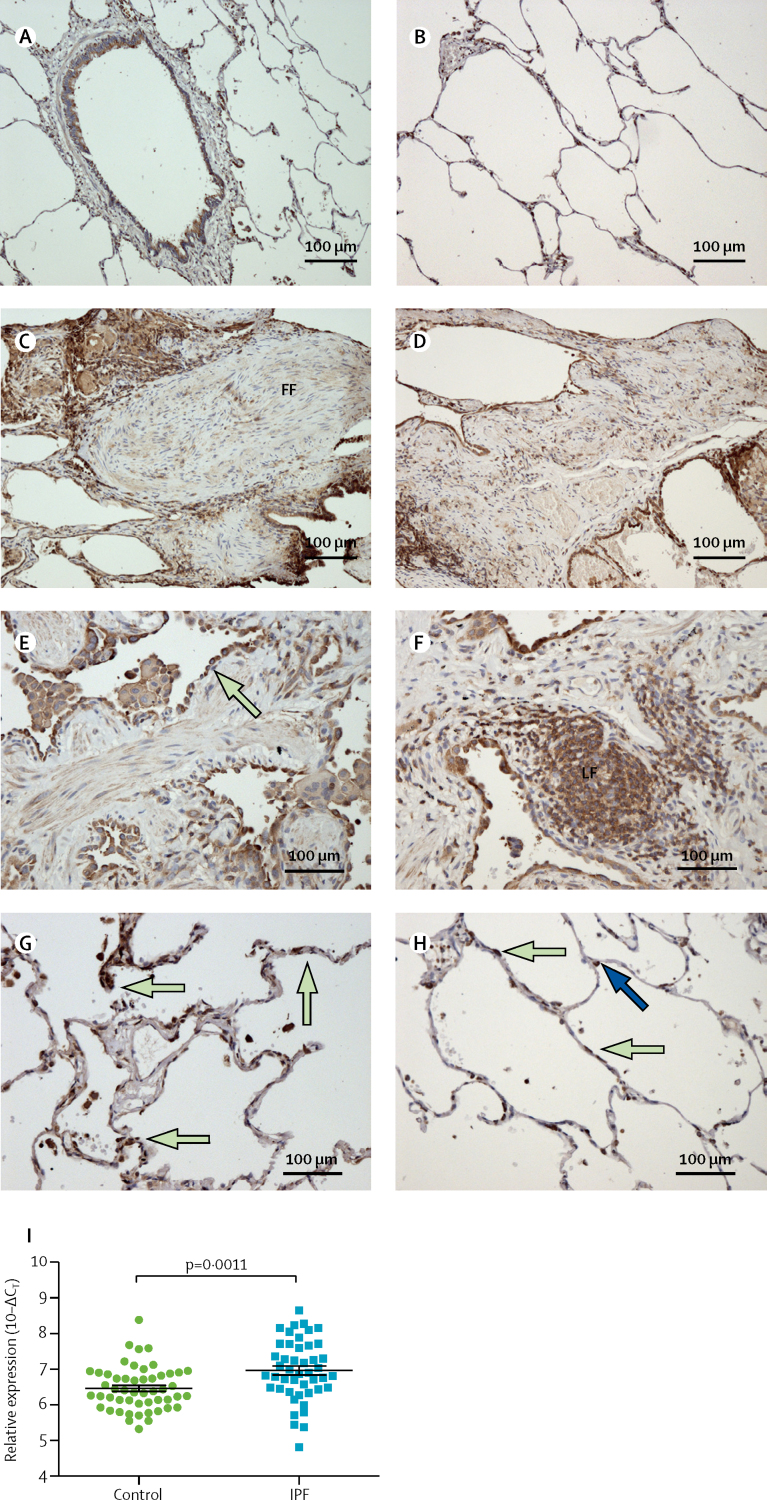
AKAP13 expression in bronchial mucosa and alveolar cells in patients with IPF and controls Sections of lung tissue from controls show AKAP13 expression in the bronchial mucosa (A) and alveolar cells (B). Sections of lung tissue from patients with IPF show low AKAP13 expression in fibroblastic foci (C), and high expression in the epithelium lining fibrotic alveoli (D) and distal small airways (green arrow; E). (F) Strong staining in lymphoid follicles associated with fibrotic regions in patients with IPF. (G) Section of lung tissue from a patient with IPF showing that areas of lung less affected by fibrosis have high numbers of alveolar cells expressing AKAP13 (green arrows). (H) In tissue from regions of the lung unaffected by fibrosis in patients with IPF, type 1 (green arrows) and type 2 (blue arrow) alveolar epithelial cells primarily express AKAP13 in the parenchyma. (I) *AKAP13* mRNA expression in whole lung tissue homogenates from patients with IPF and controls. Each point shows a sample from one person and the line shows the mean fold change (black bars show ± SE) in *AKAP13* mRNA in 46 patients with IPF and 51 controls. Relative expression (relative to housekeeping gene) is plotted on a log_2_ scale. AKAP13=A-kinase anchoring protein 13. FF=fibroblastic foci. IPF=idiopathic pulmonary fibrosis. LF=lymphoid follicle.

Having identified that the minor allele A of rs62025270 was associated with increased susceptibility to IPF and increased expression of *AKAP13* in lung tissue, we did a further analysis in lung tissue to identify the cellular location of AKAP13 in the lung and compared levels of *AKAP13* mRNA from patients with IPF versus controls. Morphological assessment of histological sections from 10 control and 10 patients with IPF showed that AKAP13 protein was expressed primarily in bronchial epithelium (figure 5A) and alveolar type 1 and 2 cells (figure 5B) in control lung samples. High AKAP13 expression was observed in fibrotic regions of lung tissue from patients with IPF, and the cells expressing AKAP13 were primarily epithelial cells, macrophages, and lymphoid aggregates (figure 5C–F). High AKAP13 expression was also seen in epithelial cells from less fibrotic alveolar regions of lungs of patients with IPF, with more AKAP13-expressing cells in alveoli of patients with IPF (figure 5G) than in alveoli of controls (figure 5H). Real-time PCR analysis of samples from 46 patients with IPF and 51 controls established that concentrations of *AKAP13* mRNA were 1·42-times higher in whole lung tissue homogenates from patients with IPF than in lung tissue from controls (p=0·0011; figure 5I). Linear regression analysis confirmed that the significant increase in *AKAP13* mRNA expression in lung tissue of patients with IPF was maintained after controlling for age, sex, and smoking history (p=2·03 × 10^−4^).

We tested the association of rs62025270 with survival time in all patients from stage 1 for whom survival data were available (n=565; 360 with recorded deaths and 205 with censored survival times, with a minimum follow-up time of 1112 days before censoring). The median survival time after diagnosis of IPF was 1621 days (4·4 years; IQR 764–2815 days). rs62025270 showed no association with survival time (hazard ratio 1·01 [95% CI 0·86–1·19], p=0·878).

11 proteins that interact with AKAP13 were identified as known targets for existing drugs or compounds in development, including aspirin, dextromethophan, and GSK-690693 (appendix pp 15–17).

## Discussion

We studied 2760 patients with IPF and 8561 controls and identified a novel genome-wide significant association of variant rs62025270 that implicates *AKAP13* as a potential driver of IPF pathogenesis. We showed that the minor allele (A) of rs62025270 (minor allele frequency [MAF]=25%), associated with increased susceptibility to IPF, was associated with increased expression of *AKAP13* in lung tissue. Morphological analysis of control and diseased lung tissue identified *AKAP13* expression primarily in epithelial cells, with some additional expression in lymphoid tissue in patients with IPF. Furthermore, we showed increased expression of *AKAP13* in fibrotic lung tissue from patients with IPF compared with samples from healthy controls.

The high AKAP13 expression in epithelial cells further supports the hypothesis that IPF is a disease characterised by epithelial susceptibility to injury, consistent with findings of other genetic risk factors associated with epithelial cell function.^13^ AKAP13 is a RhoA guanine nucleotide exchange factor (RhoGEF) that is crucial for murine cardiac development. *AKAP13*-null mice are embryonic lethal because of arrested cardiac development at embryonic day 10.^24^ Conversely, enhanced AKAP13 signalling has been shown to promote profibrotic signals in cardiac fibroblasts^25^ and leads to cardiac hypertrophy in mice.^26^ Although AKAP13 has not previously been implicated in the pathogenesis of IPF, it plays a key role in profibrotic signalling pathways. Thrombin and lysophosphatidic acid have been identified as key profibrotic mediators acting via a Gq and RhoA signalling pathway in epithelial cells to promote αvβ6 integrin-mediated transforming growth factor β (TGFβ) activation.^27^, ^28^ AKAP13 coordinates thrombin and lysophosphatidic acid receptor signalling through Ga12 to RhoA in fibroblasts^29^ and astrocytes,^30^ and has also been shown to functionally interact with Gq, promoting RhoA activation.^31^, ^32^, ^33^ Therefore, AKAP13 might promote epithelial αvβ6 integrin-mediated TGFβ activation in response to epithelial injury, thereby promoting IPF. A phase 2 clinical trial assessing the safety and tolerability of a humanised monoclonal antibody directed against the αvβ6 integrin heterodimer (compound BG00011, formerly known as STX-100) in participants with IPF is underway (NCT01371305).

We also identified AKAP13 in lymphoid follicles within fibrotic regions of lung tissue from patients with IPF. Lymphoid follicles without germinal centres are well described in IPF and are thought to be composed primarily of B cells and dendritic cells.^34^, ^35^, ^36^, ^37^, ^38^ Increased concentrations of plasma B lymphocyte stimulating factor have been reported in patients with IPF compared with either controls or patients with chronic obstructive pulmonary disease, and higher concentrations were associated with severe disease.^39^ Furthermore, analysis of gene expression profiles from lymphoid follicles identified them as the source of C-X-C motif chemokine ligand 13,^38^ a biomarker of severe IPF,^38^, ^40^ suggesting that lymphoid follicles might play an important undefined role in the pathogenesis of IPF. The role of AKAP13 in B-cell function is similarly poorly understood, although it has been shown to be responsible for glucocorticoid responsiveness in lymphocytes stimulated with lysophosphatidic acid,^41^ and is involved in lymphocyte responses to osmotic stress.^42^

Because of the known function of AKAP13, it probably has some potential downstream molecular interactions that could be targeted therapeutically, most notably RhoA and Rho kinase.^43^ 11 proteins that interact with AKAP13 were identified as known targets for existing drugs or compounds in development (appendix pp 15–17). One of the existing drugs was aspirin, which targets prostaglandin-endoperoxide synthase 1 (PTGS1) and PTGS2, and PTGS2 has a well described role in the pathogenesis of IPF.^44^, ^45^, ^46^, ^47^ Another of the drugs was dextromethorphan, a well known cough-suppressant that antagonises N-methyl-D-aspartate receptors.^48^ Notably, a novel pan AKT inhibitor, GSK-690693, was also identified as a possible asset for targeting AKAP13 binding partners, and the AKT pathway is emerging as an important pathway in fibrogenesis.^49^

To our knowledge this is the first GWAS implicating *AKAP13* at genome-wide significance in IPF susceptibility.^12^ Additionally, we confirmed seven previously reported associations with IPF susceptibility: the T allele of SNP rs35705950 in the *MUC5B* promoter (stage 1 p=1·86 × 10^−37^) was the strongest genetic risk factor for susceptibility to IPF with an OR of 4·11,^50^ then *DSP* (stage 1 p=4·14 × 10^−12^), *MUC2* (stage 1 p=1·33 × 10^−5^), *TOLLIP* (stage 1 p=1·60 × 10^−5^), *TERT* (stage 1 p=8·25 × 10^−5^), *DPP9* (stage 1 p=4·17 × 10^−4^), and *FAM13A* (stage 1 p=0·002). 17 variants were identified in stage 1 that have not been previously reported to be associated with IPF (of which 14 were low frequency or rare), but we could not investigate all of these in the stage 2 analyses because not all data were available. These could be additional true positive signals of association with susceptibility to IPF that will reach genome-wide significance in larger and more densely imputed studies.

A strength of our study is that we were able to bring together the largest sample size of patients with IPF and controls available to date and apply a robust and widely used two-stage study design^10^, ^11^, ^51^ to identify a novel genetic signal of association with IPF susceptibility. Furthermore, our stage 1 study samples were genotyped using the Affymetrix Axiom UK BiLEVE and UK Biobank arrays, which are optimised for imputation of individuals with European ancestry, and were genome-wide imputed using the most comprehensive imputation panel resource available at the time. This enabled analysis of 13 076 821 variants genome-wide with MAF of more than 0·1%.

This study also has some limitations. Although clinical investigators were careful to exclude other fibrotic lung diseases, a small number of non-IPF cases of pulmonary fibrosis might have been included. However, the advantage of collating a large dataset is that the effects of misclassification on the analysis are reduced. Furthermore, we expect that misclassification would lead to attenuation of signals rather than produce false-positive findings, which was supported by our replication of nine previously reported signals of association with IPF in our previously unreported stage 1 data. This included five signals that reached a Bonferroni-adjusted significance threshold for 16 tests (p<3·13 × 10^−3^), and two additional signals that reached genome-wide significance (*MUC5B* p=1·86 × 10^−37^ and *DSP* p=4·14 × 10^−12^). In addition, the diagnostic practices between the UK and USA might be different but the concordant results from the three case-control studies reassures us that these observations are robust.

A previous study of 119 patients with IPF and 50 donor lung controls from the Lung Tissue Resource Consortium reported a small decrease of AKAP13 expression in patients with IPF compared with controls using gene expression data from the Affymetrix Gene ST1·0 array;^52^ whereas our data using RT-PCR showed a moderate increase of *AKAP13* expression in patients with IPF compared with controls. The discordance between these results could be due to a number of factors, including the difference in normalisation strategies between the two techniques and sample heterogeneity. The eQTL data that we present from control lung tissue shows that the minor allele A of rs62025270, associated with susceptibility to IPF, is also associated with increased expression of *AKAP13* in three independent studies.^53^, ^54^, ^55^ The immunohistochemistry results provide evidence that the predominant cell types expressing AKAP13, in both normal and fibrotic lung, are epithelial cells and resident immune cells, whereas the dense fibrotic regions of lung with an expanded population of myofibroblasts (figure 5C–F) do not express AKAP13. Furthermore, eQTL data from whole blood reveals the minor allele A of rs62025270 is associated with reduced expression of *AKAP13* mRNA. Therefore, these data provide strong independent evidence that increased AKAP13 expression in alveolar cells has a role in IPF, and the cellular heterogeneity within the fibrotic lung samples used for RNA analysis is likely to explain this discordance.

In summary, we report a novel genome-wide significant association for IPF susceptibility with SNP rs62025270 and present evidence that this SNP might exert its effect via expression of the nearby gene *AKAP13*. AKAP13 is a RhoGEF that is known to interact with a central fibrogenic pathway—G protein-coupled receptor activation of RhoA—and expression is increased in fibrotic regions of lungs from patients with IPF. Expression of *AKAP13* occurs primarily in the epithelium thereby reinforcing the idea that epithelial processes are central to IPF pathogenesis. The identification of a RhoGEF that regulates a pathway known to be involved in the pathogenesis of IPF, a pathway for which drugs are in development, could potentially provide a novel target for antifibrotic therapy for IPF.
