# Supplementary material for: Genetic variants associated with susceptibility to idiopathic pulmonary fibrosis in people of European ancestry: a genome-wide association study
Source: Lancet Respir Med. 2017 Nov;5(11):869–80. doi: 10.1016/S2213-2600(17)30387-9 (PMC5666208; doi:10.1016/S2213-2600(17)30387-9)
Supplement: Supplementary appendix [file mmc1.pdf]

THE LANCET
Respiratory Medicine

Supplementary appendix

This appendix formed part of the original submission and has been peer reviewed. We post it as supplied by the authors.

Supplement to: Allen R J, Porte J, Braybrooke R, et al. Genetic variants associated with susceptibility to idiopathic pulmonary fibrosis in people of European ancestry: a genome-wide association study. *Lancet Respir Med* 2017; published online Oct 20. http://dx.doi.org/10.1016/S2213-2600(17)30387-9.

Appendix

Stage 1 case selection and quality control

In total 677 IPF cases were selected from UK IPF patient cohorts with diagnoses being made in accordance with contemporaneously accepted international criteria[1,2]. Cases were recruited from nine different centres across the UK; the Trent Lung Fibrosis (TLF) study based in Nottingham (n = 237), the PROFILE[3] (Prospective Observation of Fibrosis in the Lung Clinical Endpoints) study based in Nottingham (n = 176), University of Edinburgh (n = 134), University College London (UCL) (n = 52), Papworth Hospital NHS Foundation Trust in Cambridgeshire (n = 46), University of Hull (n = 21), Southmead Hospital in Bristol (n = 5), Royal Brompton and Harefield NHS Foundation Trust in London (n = 3) and Royal Hallamshire Hospital in Sheffield (n = 3). The earliest recorded date of diagnosis was June 1996 and the latest recorded diagnosis was July 2013. The individuals recruited by UCL were historical cases and date to pre-June 1996, however it was not possible to obtain date of diagnosis for these individuals.

Only individuals passing stringent quality control (QC) measures were included in the analyses. Of the 677 IPF cases sent for genotyping eight failed quality control measures applied by Affymetrix (one individual removed for Affymetrix Dish QC and another seven for having a call rate less than 97%). Six individuals were removed from the analysis for having outlying genome-wide heterozygosity. Following IBD (Identical by Descent) analysis, 34 individuals were removed as they were duplicates of, or related to, another individual. Six individuals were removed for having either the first or second principal component more than five standard deviations away from the mean. Finally, 21 individuals were excluded from the analysis due to either a mismatch between recorded and genetically inferred sex (n = 5), uncertainty in the diagnosis of IPF following review of the clinical data (n = 6) or missing information on age (n = 10). In summary, 602 cases passed all QC and were included in stage 1 analyses.

Selection of UK Biobank controls for stage 1

Controls were selected from UK Biobank[4] under the criteria that they must have genotype data, pass the same QC as the IPF cases, not be IPF cases or suffer from any other interstitial lung disease (ILD). Whether subjects had an ILD was checked through illnesses recorded in the UK Biobank interview process, HES (Health Episode Statistics) data and cause of death. If any of these were recorded using the ICD10 codes J84.0-J84.9 the individual was removed.

Genotyping data was available for 152,729 of the 502,682 individuals recruited by UK Biobank. Of the genotyped individuals, UK Biobank recommends removing 480 individuals for either having outlying genome-wide heterozygosity or having more than 5% missing genetic data. Those recorded as being related to any other individual in UK Biobank (n = 17,308) or not determined as being of European ancestry from the genetic data (n = 22,603) were removed. There were no sex mismatches leaving 112,338 individuals in UK Biobank passing QC. In total 148 individuals were removed as they were concluded to have an ILD leaving 112,190 individuals passing selection criteria.

Controls were selected such that they followed similar age, sex and smoking distributions to the IPF cases. The proportion of ever smokers in the IPF cases was based on those in the PROFILE study (n = 176) as this was the only centre that had smoking status data available. Accordingly, controls were selected ensuring that 70% were male, 70% were ever smokers and 83% were aged 65 or above. Five controls (plus 10% extra to allow for downstream QC failures) were selected for every IPF case passing QC meaning 3,366 controls were selected. IBD analysis between cases and controls found no additional duplicates or related individuals.

Power calculations

Power calculations were performed using Quanto[5] v1.2.4, assuming a population disease prevalence of 20 cases per 100,000 and varying effect size and effect allele frequencies. Results from these calculations can be seen in Supplementary Figure 1.

Phasing and imputation

For stage 1 samples, phasing and imputation was performed on stage 1 cases and controls together in 3Mb chunks. Phasing was performed before imputation using SHAPEIT v2 (r837)[6] using variants with a call rate greater than 95%, MAF greater than 1% and in Hardy-Weinberg equilibrium ($p > 10^{-6}$). For variants found on both the UK BiLEVE and UK Biobank array call rate was calculated across the whole sample and for variants found only on one array, call rate was calculated just in individuals genotyped using that array. Imputation was performed using IMPUTE2[7] v2.3.2 using a combined reference panel from 1000 Genomes Project[8] Phase 3 and UK10K[9].

For the Chicago Consortium, genotyping was performed using the Affymetrix Genome-Wide Human SNP 6.0 Array. Phasing and imputation used SHAPEIT[6] and minimac[10] and the HRC[11] (Haplotype Reference Consortium) r1.1 reference panel. For the Colorado Consortium the Illumina 660 Quad beadchip was used for genotyping and phasing and imputation was performed using SHAPEIT[6] and IMPUTE2[7] (using 1000 Genomes Phase 1 as the reference panel).

Variant QC

During imputation, an imputation quality score is calculated based on how certain the imputation is for each variant. Generally, imputation is of a higher quality for common variants compared to rare variants. Therefore for variants with MAF $\geq$ 1% only variants with an imputed information score ≥ 0.5 were included in the analysis and for variants with MAF < 1% only variants with an information score ≥ 0.8 were included. Due to potential biases caused by poor imputation for very rare variants, a minimum MAC threshold of 10 was set for imputed variants and a minimum MAC threshold of 3 was set for directly genotyped variants.

Stage 1 genome-wide and X chromosome analysis

Case-control analyses were run genome-wide assuming an additive genetic model conditioning on age, sex and ten principal components. Variants were recorded as dosages (i.e. continuous values between 0 and 2 to take into imputation uncertainty). The analysis was run using the score test using SNPTEST[12] v2.5.2 due to its computational efficiency. The Firth test has been shown to give a better combination of type 1 and type 2 error rate than the score test for low frequency variants (especially in unbalanced case-control studies)[13]. Therefore for variants with a score test p value $< 5 \times 10^{-3}$ and a MAC < 400 the analysis was rerun using the Firth test using EPACTS[14] v3.2.4. For the X chromosome males, the reference allele was coded as zero and the alternate allele coded as two (or the equivalent dosage) and analysed using the same approach as used for the autosomes.

Selection of signals for stage 2 analyses

Independent variants representing signals of association with susceptibility to IPF in stage 1 with $p < 5 \times 10^{-6}$ were selected for further study in stage 2. Independence of signals was confirmed using conditional analyses. Where possible, genotyping cluster plots of the most strongly associated variant of each independent signal (or a genotyped proxy, if the top variant was imputed) were manually checked and variants with evidence of poor genotype clustering were excluded from further analysis.

Accounting for array effects

The controls for stage 1 of this study were selected from UK Biobank where ~50K individuals were genotyped on the Affymetrix Axiom UK BiLEVE array (the same array as the cases) and the rest were genotyped on the 95% identical Affymetrix Axiom UK Biobank array. In order to maximise the power of our study by increasing the number of controls, we selected controls that had been genotyped on either array. As the UK Biobank controls genotyped on the Axiom UK BiLEVE array had originally been ascertained on the basis of lung function and smoking behaviour, it was reasonable to assume that allele frequency differences between the controls genotyped on the two arrays could feasibly be driven by either technical array artefacts or genuine associations with lung function and smoking. It was not possible to include an adjustment for array in the association testing model as all cases were genotyped on the same array. Furthermore, it has been shown that there is some overlap of genetic signals of lung function/COPD and pulmonary fibrosis[15]. In order to ensure that signals of association with IPF that we reported were genuine, we additionally tested for association with array by comparing controls genotyped on the UK Biobank array with controls genotyped on the UK BiLEVE array. All variants that showed association with susceptibility to IPF in stage 1were selected for analysis in stage 2. We report as genuine novel signals of association with IPF, signals that were associated with susceptibility to IPF independently in both stage 1 and stage 2, and which had no evidence of association with array ($p < 0.05$) in stage 1. The genome-wide Manhattan plot and QQ plot for the control-control analysis, including the genomic inflation factor lambda, are shown in Supplementary Figure 2. For novel signals of association with IPF susceptibility, we additionally undertook sensitivity analyses restricting the control set to individuals genotyped on the same array as the cases and repeating the association analyses, and regionally re-imputing the signal using the Haplotype Reference Consortium imputation panel and repeating the association analyses.

Stage 2 samples

Stage 2 analyses were performed on individuals from two independent samples from the USA; the Chicago Consortium and the Colorado Consortium. The Chicago Consortium dataset comprised of individuals used in the stage 1 GWAS (genome-wide association study) in the study by Noth et al[16]. Briefly, these were individuals of American-European ancestry passing QC (call rate > 97%, no sex mismatches and related individuals removed) where controls were selected so they were genetically similar (based on the first four principal components) to the IPF cases. The Colorado Consortium dataset consisted of individuals as described in the Fingerlin at al GWAS[17,18]. Cases were self-reported non-Hispanic white individuals with an IIP and controls were selected from self-reported non-Hispanic white individuals based on IBS (Identical by State) estimates such that the controls were genetically similar to the cases. Individuals were removed if they were genetic outliers based on IBS estimates, showed sex mismatches, had genome-wide heterozygosity more than four standard deviations away from the mean or had call rates < 98%.

Stage 2 case-control analysis and meta-analyses

The analyses for the Chicago Consortium and Colorado Consortium have been described elsewhere[16-18]. The Chicago Consortium adjusted for age and sex but did not adjust for principal components because cases and controls were pre-matched using genetic similarity to the cases based on principal components analysis. The Colorado Consortium adjusted for sex and three dimensions from a multi-dimensional scaling model. Dimensions were used rather than principal components as the distribution of p values when running the analysis genome-wide was closer to the null when using dimensions. Age was not included in the model as these data were not available for controls.

These two analyses were combined using an inverse variance weighted fixed effects meta-analysis. These two analyses were then meta-analysed with the stage 1 results and any variant found to be genome-wide significant ($p < 5 \times 10^{-8}$) when meta-analysing the stage 1 and stage 2 results together was deemed to show an association with susceptibility to IPF. Variants which became less significant in the meta-analysis than in stage 1 alone were not reported as showing an association with susceptibility to IPF.

eQTL

The variants identified as associated with susceptibility to IPF as well as any variants in high LD ($r^2 > 0.8$) were investigated in three eQTL datasets; a blood eQTL database[19], the GTEx project covering multiple tissues[20,21] and a lung eQTL database[22-24]. The blood eQTL database, as described by Westra et al[19], contained eQTL data for variants showing any association with peripheral blood samples from 5,311 individuals. Individuals were genotyped on various platforms and imputed using HapMap. The GTEx project includes eQTL data from multiple tissues from 449 donors genotyped using either the Illumina OMNI 5M or 2.5M arrays and imputed to the 1000 Genomes Project Phase 1 (version 3). Only the 44 tissues with at least 70 samples from genotyped donors were included in the lookup for the IPF associated variants. Finally, the lung eQTL database consisted non-tumour lung tissue samples from 1,111 individuals who had undergone lung resection surgery, mainly current or former smokers, genotyped on the Illumina Human1M-Duo BeadChip array. Cis-eQTLs were available for all three databases (defined as the variant being within 1Mb of the beginning of the transcription start site in the lung and blood databases and within 250kb in GTEx) and trans-eQTLs were available in the lung and blood databases. An FDR threshold of 10% was used for eQTLs in the blood and lung databases and 5% in the smaller GTEx resource.

mRNA analysis

RNA was extracted extracted from human lung tissues using the mirVana kit (Ambion/Life Technologies, Carlsbad, CA). Complementary DNA (cDNA) wasComplementary DNA (cDNA) was synthesized from 80ng RNA using SuperScript III (Life Technologies, Waltham, MA) and diluted 1 to 8 with nuclease-free water. cDNA was used with real-time polymerase chain reaction (PCR) gene expression assay for AKAP13 (Hs00180747_m1, Thermo Fisher Scientific) and normalized to glyceraldehyde phosphate dehydrogenase (GAPDH; Hs02786624_g1, Thermo Fisher Scientific) and TaqMan Fast Advanced Master Mix (Applied Biosystems, Carlsbad, CA) and Viaa7 real-time PCR system (Thermo Fisher Scientific). Relative gene expression was determined through the ΔΔ cycle threshold (Ct) method and ΔCt values were calculated by subtracting the Ct value for GAPDH from the Ct value for AKAP13 for a sample. All statistical analyses were computed in GraphPad Prism (v5.01, GraphPad Software, La Jolla, California, USA) and Stata (Release 14, StataCorp., College Station, Texas, USA).

Immunohistochemistry

Lung tissue from patients with IPF was obtained either post-mortem or from lung transplant patients following informed written consent and ethical review available through the MRC Nottingham Molecular Pathology Node (ethical approval numbers: Nottingham Respiratory Research Unit, 08/H0407/1; Papworth Hospital Research Tissue Bank (REC) 08/H0304/56). Non-fibrotic human lung tissue was obtained from non-cancerous tissue removed during surgery or from donor lungs unsuitable for transplant. All experiments were performed in accordance with the World Medical Association Declaration of Helsinki. Antigens were unmasked by microwaving in 10mmol citrate buffer for 20 minutes. Endogenous peroxidase activity was quenched by incubating in 3% H2O2 in methanol for 30 minutes. Sections were then incubated for one hour in 5% goat serum in PBS. The sections were incubated in primary antibody solution (anti AKAP13 rabbit polyclonal antibody – Sigma HPA019773, 1:400 dilution, in 5% goat serum blocking buffer) overnight followed by incubation in secondary anti-rabbit antibody (1:200 goat anti-rabbit – Vector BA-1000) for 30 minutes. Streptavidin label was attached by incubation in ABC complex solution (Vector PK-6100) for 30 minutes. Colour development was performed using 3,3'-diaminobenzidine tetrachloride (Sigma D4418), and counterstained using Mayer's haematoxylin (Raymond Lamb Ltd).

Signal refinement using Bayesian fine-mapping

We estimated 95% credible sets (a set of variants that is 95% likely to contain the causal variant). To do this the posterior probabilities for each variant being causal was calculated using approximate Bayes factors (ABFs) proposed by Wakefield[25]. The ABFs were calculated using the following formula:

$$\mathrm{ABF} = \frac{1}{\sqrt{1 - \frac{W}{V + W}}} \exp\left(-\frac{Z^2}{2}\frac{W}{V + W}\right)$$

where Z is the Z statistic (i.e. effect size estimate divided by the standard error of the estimate), V is the variance of the effect size and prior W. Setting a prior of $W = 0.4$ is equivalent to a 95% belief that the departure from the null model for the relative risk is less than 1.5 [26]. This formula gives the Bayes factor comparing the null hypothesis to the alternative model, so the Bayes factor for the whether a variant has an effect on the phenotype compared to the null model would be equal to the reciprocal of this formula. These were calculated using a prior of $W = 0.4$ for all variants with a stage 1 p value < 0.001 and within 1Mb of the sentinel variant.

An approximate posterior probability of the variant being causal is equal to the ABF for that variant divided by the sum of all ABFs in that signal (as the posterior probability will be proportional to the Bayes factor and the sum of the probabilities must equal one). This assumes that there is a single causal variant in the signal and that it has been included in the analysis. The credible set can be constructed by adding the variants with the highest probabilities to the credible set until the sum of the probabilities of the variants in the set exceed 0.95.

The credible set for the signal on chromosome 15 contained 113 variants and covered a region of the genome that included the *AKAP13* and *KLHL25* genes (Supplementary Table 3), the credible set for the *MUC5B* signal only contained rs35705950 and the credible set for the *DSP* signal only contained rs2076295.

Druggability

Proteins that interact with the proteins identified as associated with susceptibility to IPF were identified. This was performed using STRING[27] setting conditions that the interactions had to have "known interactions" (identified from either experiments or curated pathway databases) and only including the interactions with the "highest confidence" (confidence score > 0.9). The protein IDs (obtained from the protein database UniProt[28]) that were identified by STRING were searched in the drug databases ChEMBL[29] and DrugBank[30].

53 protein interactions with AKAP13, 18 proteins interactions with DSP and one with MUC5B were identified (Supplementary Table 7). Of these, 11 of the protein interactions with AKAP13 were returned as possible drug targets (Supplementary Table 8).

Supplementary Figures and Tables

Supplementary Figure 1: Power of the stage 1 GWAS. This plot shows the power (y axis) of the discovery case-control analysis for different allele frequencies (x axis) for 4 different effect sizes.

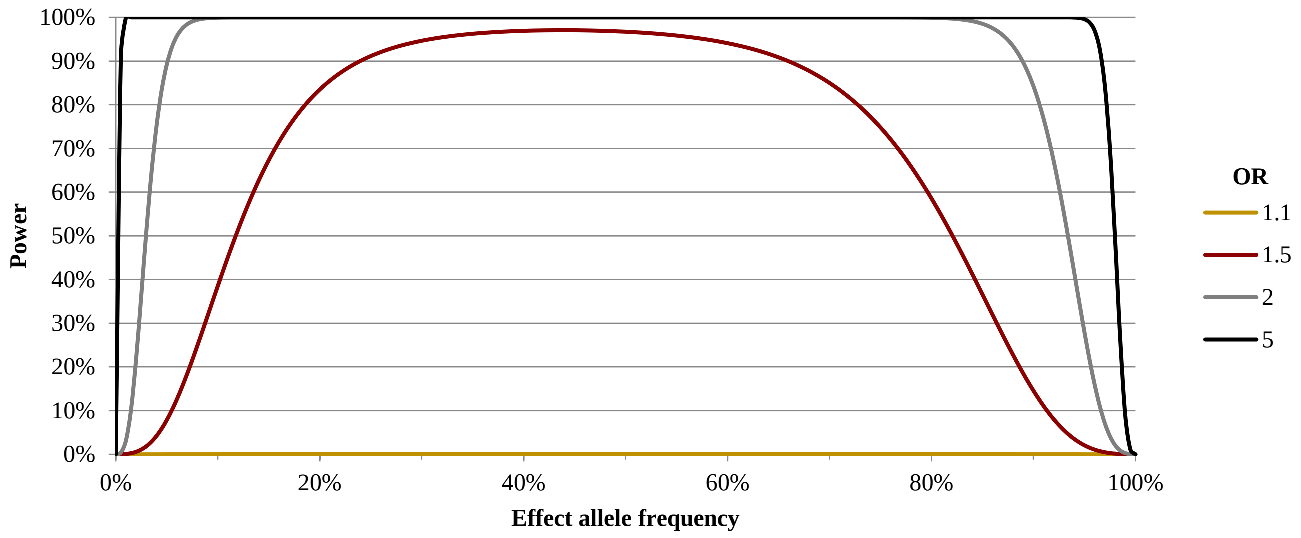

Supplementary Figure 2: Genome-wide results of control-control analysis to identify associations with array. A) Manhattan plot with position on the x axis and −log(p) value from the control-control analysis on the y axis.The blue line shows $p = 5 \times 10^{-6}$ and the red line shows $p = 5 \times 10^{-8}$. Variants in green are variants that had $p < 5 \times 10^{-6}$ in both the case-control analysis and control-control analysis B) Quantile-quantile (QQ) plot: the genomic inflation factor was 1.012.

A)
s

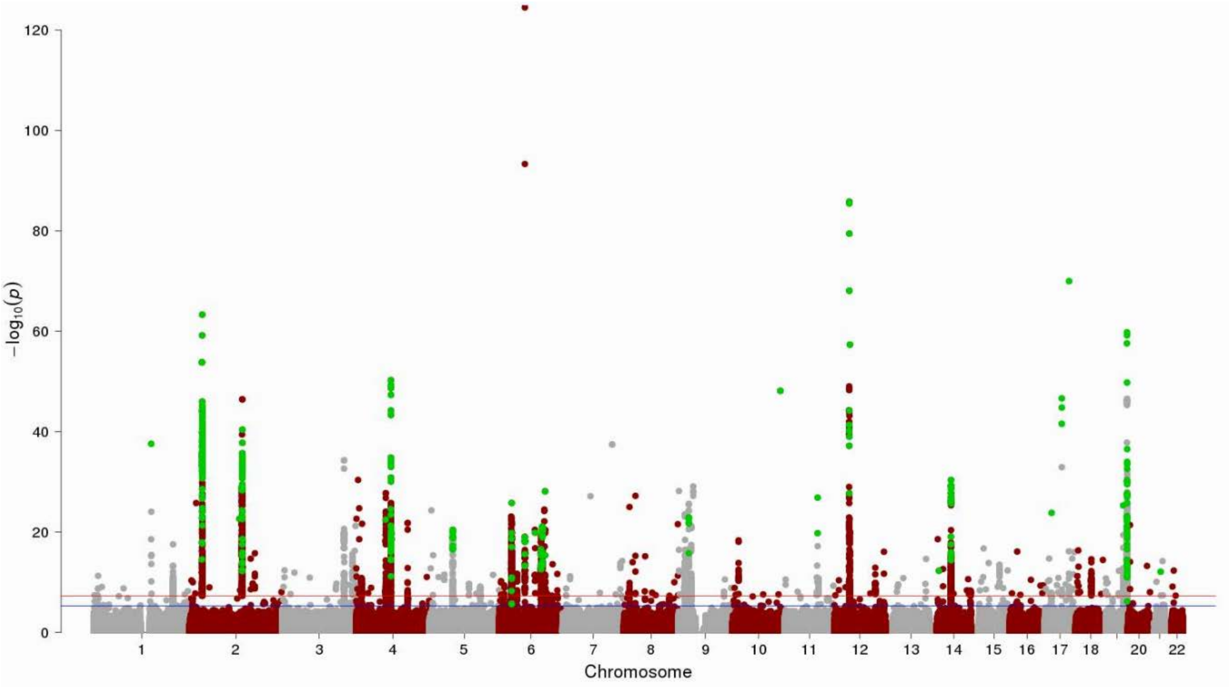

B)

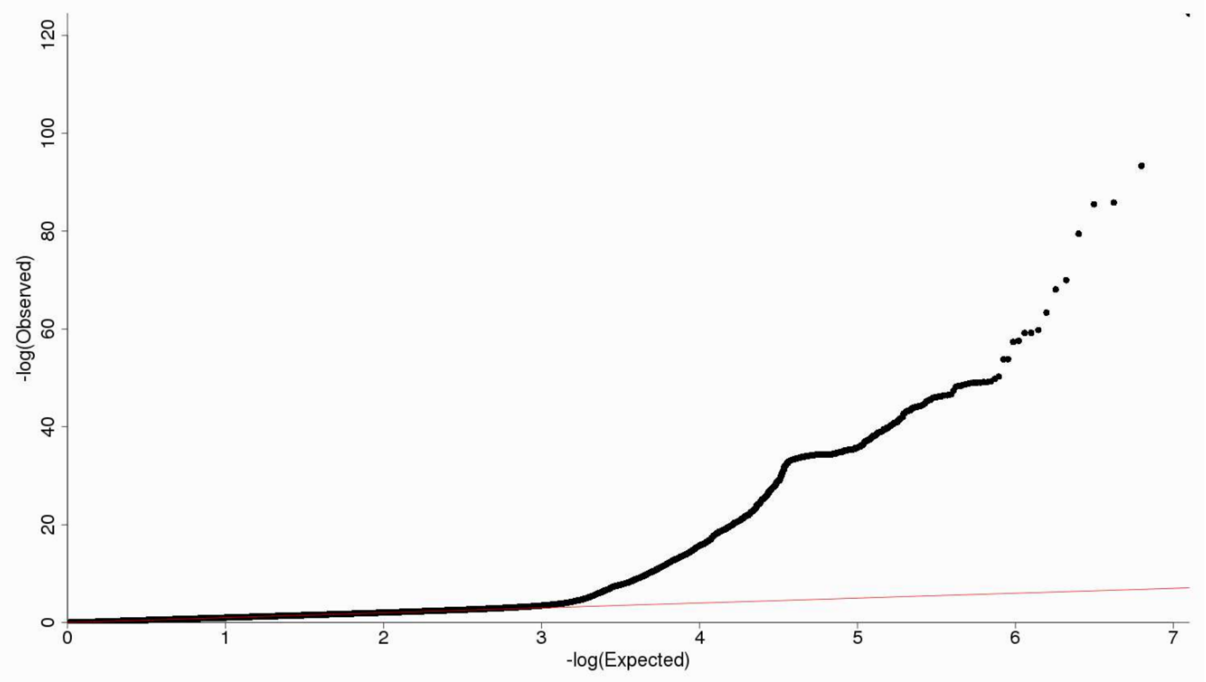

Supplementary Figure 3: Comparison of case-control results and blood eQTL results. These figures show regions plots for the discovery GWAS (circles above the x axis) and eQTL analysis (squares below the axis). The x axis shows chromosomal position. The y axis above the x axis shows the $-\log_{10}$(p value) from the case-control analysis and the y axis below the x axis shows the $-\log_{10}$(p value) for expression of *AKAP13* from the blood eQTL analysis. The blue dotted line shows the significance threshold ($p = 5 \times 10^{-6}$) used in stage 1 and the red dotted line shows genome-wide significance ($p = 5 \times 10^{-8}$). Variants are coloured by LD with rs62025270 and the genes in the region are shown at the bottom with *AKAP13* gene in green.

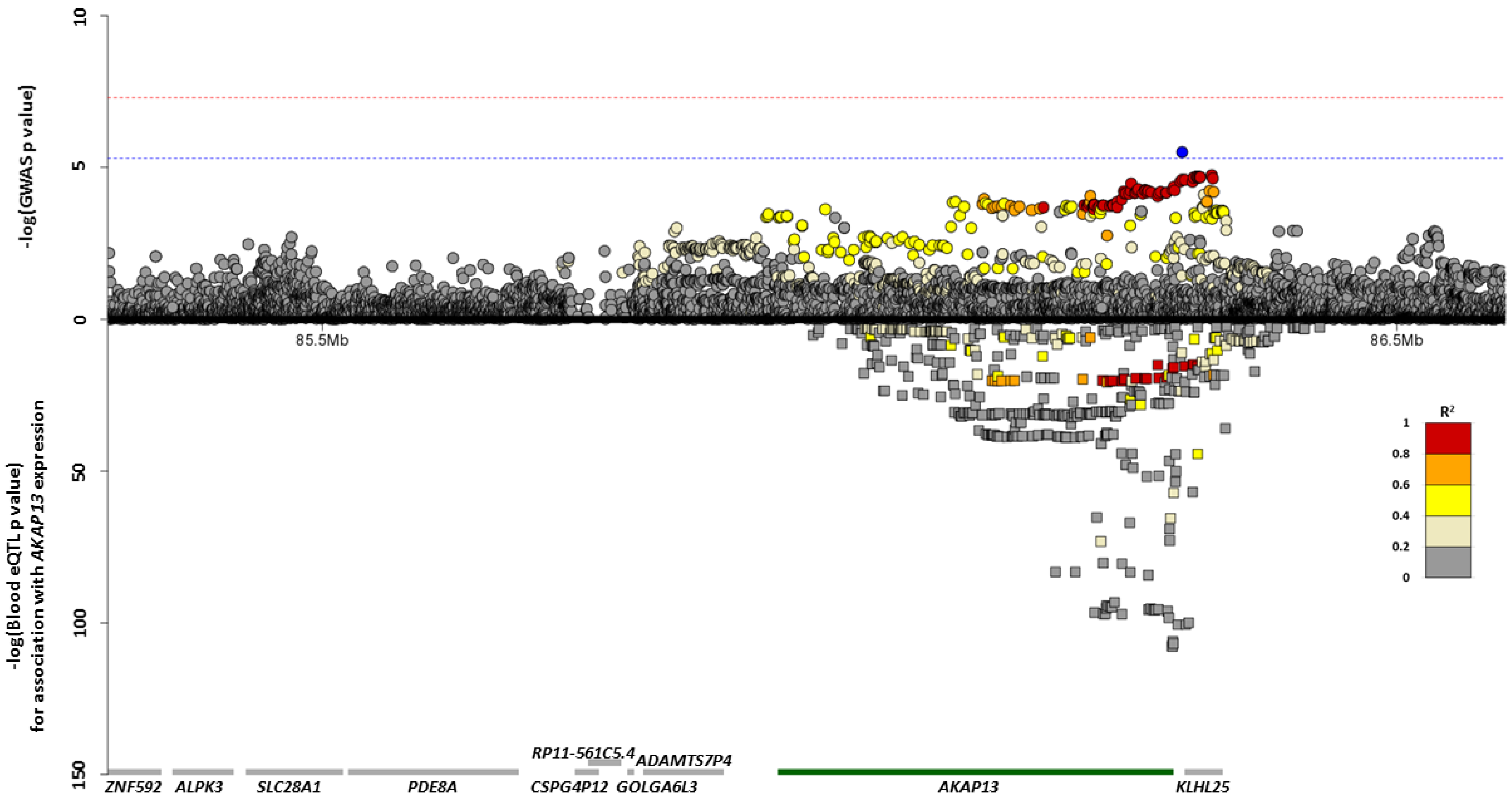

Supplementary Table 1: Summary of results for variants associated with susceptibility to IPF in the stage 1 discovery GWAS ($p < 5 \times 10^{-6}$). MAF is taken from the stage 1 discovery GWAS. Odds ratios (OR) were calculated using the minor allele as the effect allele. Stage 2 OR and p values are for the two Chicago and Colorado Consortia results meta-analysed together. Dashes (-) indicate these data were not available. Control-control P values and imputation quality are presented in Supplementary Table 4.

Chr	Position	Variant	Locus	Minor allele	Major allele	MAF	Stage 1		Stage 2		Meta-analysis (stage 1 + stage2)	
							**OR [95% CI]**	**p**	**OR [95% CI]**	**p**	**OR [95% CI]**	**p**
1	158266377	rs147562345	*CD1C*	T	C	0.9%	4.45 [2.47, 8.05]	2.90×10^{-6}	0.41 [0.10, 1.62] [b]	0.169 [b]	3.06 [1.78, 5.28]	5.34×10^{-5}
1	196625450	chr1:196625450	*CFH*	A	AAAAG	2.1%	3.27 [2.12, 5.04]	5.09×10^{-7}	-	-	-	-
2	31786481	rs191031841	*SRD5A2*	T	G	4.1%	3.07 [2.21, 4.27]	1.80×10^{-10}	0.95 [0.68, 1.32]	0.744	1.73 [1.36, 2.18]	5.58×10^{-6}
2	32853582	rs6726541	*TTC27*	G	T	5.3%	3.78 [2.62, 5.46]	1.42×10^{-12}	0.89 [0.68, 1.17]	0.394	1.48 [1.19, 1.85]	4.10×10^{-4}
2	136787476	rs74266324	2q21.3	A	G	8.6%	0.45 [0.34, 0.59]	4.56×10^{-9}	0.92 [0.82, 1.04]	0.178	0.82 [0.74, 0.92]	3.70×10^{-4}
2	136817252	rs33938858	2q22.1	C	CT	3.9%	0.20 [0.10, 0.39]	3.61×10^{-9}	-	-	-	-
2	138817685	chr2:138817685	2q22.1	G	A	0.2%	47.8 [6.90, 332]	1.65×10^{-6}	-	-	-	-
4	77154761	rs34369701	*FAM47E*	A	G	3.9%	0.29 [0.16, 0.54]	3.73×10^{-6}	0.91 [0.79, 1.05] [c]	0.210 [c]	0.86 [0.75, 0.99]	0.034
4	90576890	rs28673968	4q22.1	C	T	12.3%	0.47 [0.38, 0.58]	2.04×10^{-12}	0.88 [0.80, 0.97]	0.009	0.79 [0.72, 0.86]	1.28×10^{-7}
4	91027241	rs112863361	4q22.1	A	G	2.1%	0.022 [0.001, 0.36]	1.60×10^{-9}	0.99 [0.82, 1.20]	0.951	0.98 [0.81, 1.18]	0.806
5	60270618	rs140507420	*NDUFAF2*	G	GTGTT	2.5%	2.98 [1.98, 4.48]	5.64×10^{-7}	-	-	-	-
5	166605924	rs150626307	5q34	A	G	0.1%	154 [21.0, 1,136]	2.81×10^{-7}	-	-	-	-
6	7563232	rs2076295	*DSP*	G	T	46.3%	1.67 [1.44, 1.92]	4.14×10^{-12}	1.39 [1.29, 1.50]	2.47×10^{-18}	1.44 [1.35, 1.54]	7.81×10^{-28}
6	32269487	rs9268159	*C6orf10*	T	A	9.9%	0.43 [0.32, 0.58]	2.19×10^{-8}	-	-	-	-
6	66932852	rs208501	6q12	T	C	16.4%	1.83 [1.51, 2.22]	8.45×10^{-10}	0.96 [0.86, 1.07]	0.496	1.12 [1.02, 1.23]	0.017
6	112115355	rs1409839	*FYN*	C	T	9.4%	0.53 [0.42, 0.67]	1.10×10^{-7}	1.07 [0.96, 1.19]	0.183	0.95 [0.87, 1.05]	0.342
8	121446432	chr8:121446432	*MRPL13*	T	C	0.3%	18.1 [6.07, 53.9]	6.69×10^{-7}	4.39 [0.14, 135] [b]	0.247 [b]	15.9 [5.61, 45.0]	1.90×10^{-7}
9	24010289	rs10117639	9p21.3	C	G	0.2%	25.3 [6.47, 98.8]	4.01×10^{-6}	1.52 [0.89, 2.58]	0.122	2.20 [1.34, 3.61]	0.002
9	27446846	rs118048878	*MOB3B*	A	G	1.8%	0.028 [0.002, 0.45]	1.50×10^{-7}	0.86 [0.64, 1.17]	0.349	0.83 [0.61, 1.12]	0.227
9	27499407	rs1975503	*MOB3B*	C	T	2.3%	0.15 [0.05, 0.44]	1.27×10^{-6}	1.00 [0.83, 1.21]	0.997	0.94 [0.79, 1.14]	0.544
10	65359362	chr10:65359362	*REEP3*	T	TTC	0.2%	36.4 [7.75, 172]	1.16×10^{-6}	-	-	-	-
10	77228877	chr10:77228877	10q22.2	A	C	0.2%	21.1 [6.25, 70.9]	7.53×10^{-7}	-	-	-	-
10	107751048	chr10:107751048	10q25.1	C	T	0.3%	27.9 [7.21, 108]	6.44×10^{-7}	-	-	-	-
11	1241221	rs35705950	*MUC5B*	T	G	14.3%	4.11 [3.31, 5.11]	1.86×10^{-37}	2.46 [2.13, 2.85]	3.13×10^{-34}	2.89 [2.56, 3.26]	1.12×10^{-66}
11	43823527	chr11:43823527	*HSD17B12*	A	AT	27.8%	1.47 [1.25, 1.72]	2.53×10^{-6}	-	-	-	-
12	38910545	rs181297970	12q12	T	G	1.1%	6.18 [3.17, 12.0]	3.27×10^{-7}	-	-	-	-
12	38945784	chr12:38945784	12q12	T	A	2.3%	3.84 [2.44, 6.03]	2.08×10^{-8}	-	-	-	-
13	24044887	rs186638373	*LINC00327*	C	A	0.1%	131 [14.4, 1,192]	5.52×10^{-7}	0.98 [0.01, 95.0] [c]	0.993 [c]	51.8 [7.09, 378.7]	1.00×10^{-4}
13	97652906	rs193268061	13q32.1	C	T	0.1%	49.3 [7.76, 313]	3.46×10^{-6}	1.37 [0.22, 8.45] [c]	0.728 [c]	7.98 [2.18, 29.1]	0.002
14	55385305	rs76271340	14q22.2	T	G	8.9%	0.48 [0.37, 0.61]	1.15×10^{-8}	0.95 [0.77, 1.17]	0.630	0.72 [0.61, 0.84]	5.34×10^{-5}
15	86300198	rs62025270	*AKAP13/KLHL25*	A	G	24.7%	1.49 [1.26, 1.76]	3.11×10^{-6}	1.22 [1.11, 1.33]	9.96×10^{-6}	1.27 [1.18, 1.37]	1.32×10^{-9}
16	84035	rs367849850	*IL9RP3*	G	[a]	3.9%	2.38 [1.68, 3.35]	1.82×10^{-6}	-	-	-	-
17	45007748	rs117791180	*GOSR2*	T	C	3.4%	2.81 [1.96, 4.01]	7.87×10^{-8}	0.92 [0.52, 1.62]	0.763	2.04 [1.51, 2.77]	3.55×10^{-6}
19	45429708	rs60049679	*APOC1P1*	C	G	4.8%	0.35 [0.21, 0.58]	2.47×10^{-6}	0.79 [0.65, 0.96]	0.018	0.71 [0.59, 0.85]	2.33×10^{-4}
19	54931983	rs73606754	*TTYH1*	G	C	13.7%	0.46 [0.38, 0.57]	2.24×10^{-13}	0.89 [0.73, 1.07]	0.217	0.65 [0.57, 0.75]	3.27×10^{-9}
19	55224185	rs141247056	*LILRP2*	A	AAC	7.3%	3.17 [2.35, 4.28]	4.49×10^{-14}	-	-	-	-
19	55557609	19:55557609:G:A	*RDH13*	A	G	0.3%	63.0 [15.4, 257]	2.25×10^{-11}	-	-	-	-
19	55567044	rs28591280	*RDH13*	C	A	8.4%	0.47 [0.36, 0.60]	2.68×10^{-9}	0.98 [0.87, 1.10]	0.718	0.85 [0.76, 0.95]	0.003
22	44250669	chr22:44250669	*SULT4A1*	T	C	0.2%	55.6 [9.50, 326]	1.28×10^{-6}	-	-	-	-

[a] The major allele is GGGGAGCCTGGAAGCACAC

[b] Analysis only run in Chicago Consortium (542 cases vs 502 controls)

[c] Analysis only run in Colorado Consortium (1,616 cases vs 4,683 controls)

Supplementary Table 2: Summary of variants that met the threshold $p < 5 \times 10^{-6}$ in stage 1only when conditioned on another variant. Odds ratios (OR) were calculated using the minor allele as the effect allele. Dashes (-) indicate these data were not available.

Chr	Position	Variant	Conditional on	Locus	Minor allele	Major allele	MAF	Stage 1		Stage 2		Meta-analysis (Stage 1+Stage 2)	
								OR [95% CI]	p	OR [95% CI]	p	OR [95% CI]	p
2	32186264	rs185218186	rs191031841	*MEMO1*	C	T	1.4%	0.14 [0.06, 0.33]	4.93×10^{-7}	1.17 [0.53, 2.58]	0.701	0.43 [0.24, 0.77]	0.005
2	136787402	rs6716536	rs74266324	2q21.3	T	C	23.1%	1.71 [1.39, 2.09]	3.26×10^{-7}	0.93 [0.82, 1.05]	0.233	1.08 [0.98, 1.20]	0.131
6	66955969	rs208524	rs208501	6q12	G	A	12.4%	0.26 [0.19, 0.38]	7.12×10^{-13}	0.48 [0.24, 0.98]	0.045	0.30 [0.22, 0.42]	2.75×10^{-13}
9	27489418	rs76324761	rs1975503	*MOB3B*	A	G	4.2%	3.73 [2.44, 5.69]	4.11×10^{-9}	0.98 [0.64, 1.50]	0.941	1.91 [1.42, 2.58]	2.23×10^{-5}
19	55201343	rs149722959	rs141247056	19q13.42	G	GT	3.7%	0.23 [0.13, 0.40]	1.33×10^{-9}	-	-	-	-

Supplementary Table 3: Variants included in 95% credible set for novel signal seen on chromosome 15.

rsid	Approximate Bayes Factor	Posterior Probability
rs62025270	5830.4	0.148
rs148937240:86327785:A:AT	1106.8	0.028
rs62025272	1006.1	0.026
rs55738258	974.7	0.025
rs56388190	971.5	0.025
rs55957626	962.7	0.024
rs62025297	962.3	0.024
rs62025298	962.0	0.024
rs17577869	940.3	0.024
rs12324721	933.4	0.024
rs66853549	892.4	0.023
rs62025269	800.3	0.020
rs2554	798.5	0.020
rs62025266	710.9	0.018
rs17639314	696.1	0.018
chr15:86252892	633.4	0.016
rs7173923	487.6	0.012
rs62022943	420.2	0.011
rs7179917	400.6	0.010
rs3169119	388.8	0.010
rs7164373	387.2	0.010
rs62022942	382.2	0.010
rs62025262	381.4	0.010
rs55968154	368.0	0.009
rs55851385	368.0	0.009
rs7182210	366.5	0.009
rs62022940	365.7	0.009
rs12148610	358.0	0.009
rs2455560	349.9	0.009
rs76902341	346.5	0.009
rs62022947	334.5	0.008
rs12148571	330.9	0.008
rs17575870	330.6	0.008
rs17576534	327.4	0.008
rs2542607	327.1	0.008
rs11073516	326.0	0.008
rs62022941	314.5	0.008
rs62022938	310.5	0.008
rs17637411	309.5	0.008
rs17638180	309.2	0.008
rs79083718:86263529:T:A	308.0	0.008
rs62022939	303.0	0.008
rs2542595	257.2	0.007
rs10163064	252.1	0.006
rs338519:86214879:T:C	251.7	0.006
rs13379832	209.7	0.005
rs7166540	203.3	0.005
rs62022926	168.3	0.004
rs2430835	165.2	0.004
rs338520	163.1	0.004
rs28515484	162.4	0.004
rs2430836	156.9	0.004
rs16941157	156.2	0.004
rs80040720	151.1	0.004
rs62022915	147.1	0.004
rs80036674	145.9	0.004
rs8035496	145.2	0.004
rs145993106:86218094:A:AAT	145.0	0.004

rs16943251	138.5	0.004
rs62022919	138.4	0.004
rs17636096	137.0	0.003
rs1026722	136.3	0.003
rs62022910	135.5	0.003
rs62022924	135.1	0.003
rs1807309	134.6	0.003
rs16943120	134.1	0.003
rs1861857	133.8	0.003
rs12324250	133.4	0.003
rs62022925	133.2	0.003
rs1861858	132.7	0.003
15:86224963:T: <INS:ME:ALU>:86225231	131.2	0.003
rs17571078	128.3	0.003
rs7181796	127.8	0.003
rs10520594	125.6	0.003
rs74025655	125.5	0.003
rs416916	124.2	0.003
rs62022920	123.9	0.003
rs6496109	123.6	0.003
rs62023935	122.9	0.003
rs140794268:86217083:AT:A	121.7	0.003
rs8029034	120.1	0.003
rs8023985	119.2	0.003
rs338536	118.5	0.003
rs78572940	118.5	0.003
rs56153788	116.0	0.003
rs62023891	115.9	0.003
rs16944522	114.2	0.003
rs55981798	112.9	0.003
rs111906684	112.5	0.003
rs7177107	111.8	0.003
rs386346	110.6	0.003
rs79719545	109.9	0.003
rs111578069:86338013:C:T	109.6	0.003
rs75890212	109.5	0.003
rs338540	109.5	0.003
chr15:86186251	109.1	0.003
rs60382493:86319403:CA:C	109.0	0.003
rs146338862	107.4	0.003
rs16944491	105.5	0.003
rs62022918	102.5	0.003
rs62022065	97.1	0.002
rs113962703	96.9	0.002
rs55639123	95.8	0.002
rs17633959	95.0	0.002
rs138560577:86191256:A:AT	94.5	0.002
rs8041288	93.1	0.002
rs75595815	91.6	0.002
rs3833025:86262129:TA:TAA	86.9	0.002
rs77768079	80.5	0.002
rs80251970	79.0	0.002
rs338517	77.1	0.002
rs2937955	76.6	0.002
rs5814216	75.9	0.002

Supplementary Table 4: Control-control comparison p values and imputation info quality scores for all variants associated with IPF susceptibility in the stage 1 discovery GWAS (see Supplementary Table 1)

Chr	Position	Variant	Locus	MAF	Imputation info	Control-control p
1	158266377	rs147562345	*CD1C*	0.9%	0.934	0.947
1	196625450	chr1:196625450	*CFH*	2.1%	0.913	0.969
2	31786481	rs191031841	*SRD5A2*	4.1%	0.816	1.59×10^{-54}
2	32853582	rs6726541	*TTC27*	5.3%	0.867	3.97×10^{-45}
2	136787476	rs74266324	2q21.3	8.6%	0.832	3.86×10^{-41}
2	136817252	rs33938858	2q22.1	3.9%	0.989	3.35×10^{-33}
2	138817685	chr2:138817685	2q22.1	0.2%	0.859	0.210
4	77154761	rs34369701	*FAM47E*	3.9%	0.828	3.16×10^{-23}
4	90576890	rs28673968	4q22.1	12.3%	0.963	5.44×10^{-51}
4	91027241	rs112863361	4q22.1	2.1%	1	1.02×10^{-21}
5	60270618	rs140507420	*NDUFAF2*	2.5%	0.893	1.49×10^{-17}
5	166605924	rs150626307	5q34	0.1%	0.839	0.642
6	7563232	rs2076295	*DSP*	46.3%	0.988	0.300
6	32269487	rs9268159	*C6orf10*	9.9%	0.766	1.83×10^{-26}
6	66932852	rs208501	6q12	16.4%	0.962	9.39×10^{-20}
6	112115355	rs1409839	*FYN*	9.4%	1	3.80×10^{-21}
8	121446432	chr8:121446432	*MRPL13*	0.3%	0.958	0.843
9	24010289	rs10117639	9p21.3	0.2%	0.865	0.524
9	27446846	rs118048878	*MOB3B*	1.8%	1	1.57×10^{-16}
9	27499407	rs1975503	*MOB3B*	2.3%	0.992	1.58×10^{-23}
10	65359362	chr10:65359362	*REEP3*	0.2%	0.814	0.850
10	77228877	chr10:77228877	10q22.2	0.2%	0.821	0.993
10	107751048	chr10:107751048	10q25.1	0.3%	0.815	0.396
11	1241221	rs35705950	*MUC5B*	14.3%	0.908	0.229
11	43823527	chr11:43823527	*HSD17B12*	27.8%	0.972	0.704
12	38910545	rs181297970	12q12	1.1%	0.668	6.36×10^{-45}
12	38945784	chr12:38945784	12q12	2.3%	0.740	3.47×10^{-80}
13	24044887	rs186638373	*LINC00327*	0.1%	0.918	0.373
13	97652906	rs193268061	13q32.1	0.1%	0.877	0.426
14	55385305	rs76271340	14q22.2	8.9%	1	2.78×10^{-18}
15	86300198	rs62025270	*AKAP13*/*KLHL25*	24.7%	0.995	0.350
16	84035	rs367849850	*IL9RP3*	3.9%	0.842	0.481
17	45007748	rs117791180	*GOSR2*	3.4%	0.818	1.53×10^{-45}
19	45429708	rs60049679	*APOC1P1*	4.8%	1	4.79×10^{-26}
19	54931983	rs73606754	*TTYH1*	13.7%	1	2.57×10^{-58}
19	55224185	rs141247056	*LILRP2*	7.3%	0.917	1.62×10^{-60}
19	55557609	19:55557609:G:A	*RDH13*	0.3%	0.850	6.09×10^{-7}
19	55567044	rs28591280	*RDH13*	8.4%	0.981	5.07×10^{-28}
22	44250669	chr22:44250669	*SULT4A1*	0.2%	0.808	0.316

Supplementary Table 5: Stage 1 association results for previously reported IPF susceptibility loci. Results from the discovery GWAS for 16 independent signals previously reported as associated with susceptibility to IPF in the previous GWAS studies of Mushiroda et al[31], Noth et al[16] and Fingerlin et al[17,18]. * indicate variants that reach a Bonferroni corrected significance threshold for 16 tests ($p < 3.125 \times 10^{-3}$). The minor allele is also the coded allele.

Chromosome	SNP	Minor Allele	MAF	Location	Where previously reported	OR [95% CI]	p	Direction of effect consistent with previous report
3	rs6793295	C	27.1%	*LRRC34*	Fingerlin et al (2013)	1.20 [1.02, 1.41]	0.032	Yes
4	rs2609255	G	22.1%	*FAM13A*	Fingerlin et al (2013)	1.31 [1.10, 1.56]	0.002 *	Yes
5	rs2736100	A	48.8%	*TERT*	Mushiroda et al, Fingerlin et al (2013)	1.33 [1.15, 1.53]	8.25×10^{-5} *	Yes
6	rs2076295	G	46.3%	*DSP*	Fingerlin et al (2013)	1.67 [1.44, 1.92]	4.14×10^{-12} *	Yes
6	rs7887	T	31.8%	*EHMT2*	Fingerlin et al (2016)	1.16 [0.97, 1.39]	0.106	Yes
7	rs4727443	A	39.8%	7q22.1	Fingerlin et al (2013)	1.12 [0.96, 1.30]	0.141	Yes
10	rs11191865	A	49.5%	*OBFC1*	Fingerlin et al (2013)	1.02 [0.88, 1.18]	0.836	Yes
11	rs7934606	T	44.9%	*MUC2*	Fingerlin et al (2013)	1.39 [1.20, 1.61]	1.33×10^{-5} *	Yes
11	rs35705950	T	14.3%	*MUC5B*	Noth et al, Fingerlin et al (2013)	4.11 [3.31, 5.11]	1.86×10^{-37} *	Yes
11	rs111521887 [a]	G	19.8%	*TOLLIP*	Noth et al	1.49 [1.24, 1.79]	1.60×10^{-5} *	Yes
11	rs5743890	C	14.7%	*TOLLIP*	Noth et al	0.79 [0.64, 0.97]	0.024	No
13	rs1278769	A	22.1%	*ATP11A*	Fingerlin et al (2013)	0.84 [0.70, 1.01]	0.058	Yes
14	rs7144383	G	11.7%	*MDGA2*	Noth et al	0.91 [0.73, 1.14]	0.421	No
15	rs2034650	A	47.7%	*IVD*	Fingerlin et al (2013)	1.16 [1.01, 1.34]	0.042	Yes
17	rs1981997 [b]	A	22.5%	*MAPT*	Fingerlin et al (2013)	0.86 [0.72, 1.01]	0.073	Yes
19	rs12610495	G	30.6%	*DPP9*	Fingerlin et al (2013)	1.33 [1.13, 1.55]	4.17×10^{-4} *	Yes

[a] Noth et al also reported rs5743894 in *TOLLIP* as associated with IPF however this is in strong LD with rs111521887 ($r^2 = 0.96$) and these are not independent signals. The OR from the discovery GWAS for rs5743894 was 1.49 [1.25, 1.79]

[b] Noth et al also reported rs17690703 in *SPPL2C* as associated with IPF however this is in strong LD with rs1981997 and these are not independent signals. The OR from the discovery analysis for a proxy [32] of rs17690703 (rs17769490, $r^2 = 0.786$) was 0.88 [0.74, 1.03]

Supplementary Table 6: Full results from eQTL analysis. Expression direction is for the variant that was found to increase susceptibility to IPF, for example if it reads "Decreased expression" then the variant that increased susceptibility to IPF decreased the expression of the stated gene.

Gene	Tissue Type	Data source	Expression direction	p
**rs35705950**				
MUC5B	Lung	GTEx	Increased expression	2.86×10^{-11}
**rs2076295**				
DSP	Blood	Blood eQTL database	Decreased expression	6.33×10^{-7}
	Lung	Lung eQTL database	Decreased expression	4.62×10^{-124}
		GTEx	Decreased expression	8.18×10^{-31}
RP3-512B11.3	Lung	GTEx	Decreased expression	7.69×10^{-6}
**rs62025270**				
AKAP13	Blood	Blood eQTL database	Decreased expression	3.01×10^{-16} [a]
	Lung	Lung eQTL database	Increased expression	1.09×10^{-17}
RP11-158M2.3	Brain (caudate basal ganglia)	GTEx	Decreased expression	9.35×10^{-7}
	Testis	GTEx	Decreased expression	2.08×10^{-8}
RP11-158M2.4	Breast (mammary gland)	GTEx	Decreased expression	4.91×10^{-6} [b]
	Nerve (tibial)	GTEx	Decreased expression	2.26×10^{-6}
	Skin (sun exposed lower leg)	GTEx	Decreased expression	6.37×10^{-7}
	Testis	GTEx	Decreased expression	7.51×10^{-8}
RP11-158M2.5	Adipose (subcutaneous)	GTEx	Decreased expression	1.68×10^{-8}
	Brain (cortex)	GTEx	Decreased expression	6.61×10^{-9}
	Brain (hypothalamus)	GTEx	Decreased expression	2.12×10^{-6}
	Lung	GTEx	Decreased expression	7.42×10^{-6} [c]
	Muscle (skeletal)	GTEx	Decreased expression	1.87×10^{-7}
	Nerve (tibial)	GTEx	Decreased expression	2.21×10^{-14}
	Skin (sun exposed lower leg)	GTEx	Decreased expression	1.79×10^{-10}
	Testis	GTEx	Decreased expression	4.25×10^{-14}
	Thyroid	GTEx	Decreased expression	3.75×10^{-7}
RP11-815J21.3	Testis	GTEx	Increased expression	5.55×10^{-27}
RP11-815J21.4	Testis	GTEx	Increased expression	4.01×10^{-6}

[a] Results from a proxy variant (rs2554, $r^2 = 0.93$)
[b] Results from a proxy variant (rs12324721, $r^2 = 0.93$)
[c] Results from a proxy variant (rs62025269, $r^2 = 0.93$)

Supplementary Table 7: List of proteins identified by STRING as interacting with variants associated with susceptibility to IPF.

Protein	UniProt ID	Protein annotation
**DSP**	**P15924**	**desmoplakin**
PKP4	Q99569	plakophilin 4
**MUC5B**	**Q9HC84**	**mucin 5B, oligomeric mucus/gel-forming**
B3GNT2	Q9NY97	UDP-GlcNAc:betaGal beta-1,3-N-acetylglucosaminyltransferase 2
B3GNT4	Q9C0J1	UDP-GlcNAc:betaGal beta-1,3-N-acetylglucosaminyltransferase 4
B3GNT7	Q8NFL0	UDP-GlcNAc:betaGal beta-1,3-N-acetylglucosaminyltransferase 7
B3GNT8	Q7Z7M8	UDP-GlcNAc:betaGal beta-1,3-N-acetylglucosaminyltransferase 8
B4GALT5	O43286	UDP-Gal:betaGlcNAc beta 1,4- galactosyltransferase, polypeptide 5
C1GALT1	Q9NS00	core 1 synthase, glycoprotein-N-acetylgalactosamine 3-beta-galactosyltransferase, 1
C1GALT1C1	Q96EU7	C1GALT1-specific chaperone 1
GALNT4	Q8N4A0	UDP-N-acetyl-alpha-D-galactosamine:polypeptide N-acetylgalactosaminyltransferase 4 (GalNAc-T4)
GALNT5	Q7Z7M9	UDP-N-acetyl-alpha-D-galactosamine:polypeptide N-acetylgalactosaminyltransferase 5 (GalNAc-T5)
GALNT6	Q8NCL4	UDP-N-acetyl-alpha-D-galactosamine:polypeptide N-acetylgalactosaminyltransferase 6 (GalNAc-T6)
GALNT8	Q9NY28	UDP-N-acetyl-alpha-D-galactosamine:polypeptide N-acetylgalactosaminyltransferase 8 (GalNAc-T8)
GALNT11	Q8NCW6	UDP-N-acetyl-alpha-D-galactosamine:polypeptide N-acetylgalactosaminyltransferase 11 (GalNAc-T11)
GALNT12	Q8IXK2	UDP-N-acetyl-alpha-D-galactosamine:polypeptide N-acetylgalactosaminyltransferase 12 (GalNAc-T12)
GALNT14	Q96FL9	UDP-N-acetyl-alpha-D-galactosamine:polypeptide N-acetylgalactosaminyltransferase 14 (GalNAc-T14)
GALNTL2	Q8N3T1	UDP-N-acetyl-alpha-D-galactosamine:polypeptide N-acetylgalactosaminyltransferase-like 2
MUCL1	Q96DR8	mucin-like 1
ST6GAL1	P15907	ST6 beta-galactosamide alpha-2,6-sialyltranferase 1
ST6GALNAC4	Q9H4F1	ST6 (alpha-N-acetyl-neuraminyl-2,3-beta-galactosyl-1,3)-N-acetylgalactosaminide alpha-2,6-sialyltransferase 4
**AKAP13**	**Q12802**	**A kinase (PRKA) anchor protein 13**
A2M	P01023	alpha-2-macroglobulin
ARAP1	Q96P48	ArfGAP with RhoGAP domain, ankyrin repeat and PH domain 1
ARAP2	Q8WZ64	ArfGAP with RhoGAP domain, ankyrin repeat and PH domain 2
ARHGAP6	O43182	Rho GTPase activating protein 6
ARHGAP11A	Q6P4F7	Rho GTPase activating protein 11A
ARHGAP11B	Q3KRB8	Rho GTPase activating protein 11B
ARHGAP12	Q8IWW6	Rho GTPase activating protein 12
ARHGAP18	Q8N392	Rho GTPase activating protein 18
ARHGAP23	Q9P227	Rho GTPase activating protein 23
ARHGAP28	Q9P2N2	Rho GTPase activating protein 28
ARHGAP30	Q7Z6I6	Rho GTPase activating protein 30
ARHGAP31	Q2M1Z3	Rho GTPase activating protein 31
ARHGAP33	O14559	Rho GTPase activating protein 33
ARHGAP36	Q6ZRI8	Rho GTPase activating protein 36
ARHGAP39	Q9C0H5	Rho GTPase activating protein 39

ARHGAP40	Q5TG30	Rho GTPase activating protein 40
CDC42	P60953	cell division cycle 42 (GTP binding protein, 25kDa)
DEPDC1B	Q8WUY9	DEP domain containing 1B
DEPDC7	Q96QD5	DEP domain containing 7
GMIP	Q9P107	GEM interacting protein
GNA12	Q03113	guanine nucleotide binding protein (G protein) alpha 12
INPP5B	P32019	inositol polyphosphate-5-phosphatase, 75kDa
MYO9A	B2RTY4	myosin IXA
MYO9B	Q13459	myosin IXB
OCRL	Q01968	oculocerebrorenal syndrome of Lowe
PRKACB	P22694	protein kinase, cAMP-dependent, catalytic, beta
PRKACG	P22612	protein kinase, cAMP-dependent, catalytic, gamma
PRKAG1	P54619	protein kinase, AMP-activated, gamma 1 non-catalytic subunit
PRKAR2A	P13861	protein kinase, cAMP-dependent, regulatory, type II, alpha
RAC1	P63000	ras-related C3 botulinum toxin substrate 1 (rho family, small GTP binding protein Rac1)
RAC2	P15153	ras-related C3 botulinum toxin substrate 2 (rho family, small GTP binding protein Rac2)
RAC3	P60763, Q9Y6Q9	ras-related C3 botulinum toxin substrate 3 (rho family, small GTP binding protein Rac3)
RHOA	P61586	ras homolog family member A
RHOB	P62745	ras homolog family member B
RHOBTB1	O94844	Rho-related BTB domain containing 1
RHOBTB2	Q9BYZ6	Rho-related BTB domain containing 2
RHOC	P08134	ras homolog family member C
RHOD	O00212	ras homolog family member D
RHOF	Q9HBH0	ras homolog family member F (in filopodia)
RHOG	P84095	ras homolog family member G
RHOH	Q15669	ras homolog family member H
RHOJ	Q9H4E5	ras homolog family member J
RHOQ	P17081	ras homolog family member Q
RHOT1	Q8IXI2	ras homolog family member T1
RHOT2	Q8IXI1	ras homolog family member T2
RHOU	Q7L0Q8	ras homolog family member U
RHOV	Q96L33	ras homolog family member V
SRGAP1	Q7Z6B7	SLIT-ROBO Rho GTPase activating protein 1
SRGAP2	O43295, O75044	SLIT-ROBO Rho GTPase activating protein 2
STARD8	Q92502	StAR-related lipid transfer (START) domain containing 8
STARD13	Q9Y3M8	StAR-related lipid transfer (START) domain containing 13
SYDE1	Q6ZW31	synapse defective 1, Rho GTPase
SYDE2	Q5VT97	synapse defective 1, Rho GTPase, homolog 2 (C. elegans)

Supplementary Table 8: Potential drug targets for proteins that interact with *AKAP13*. Evidence and pathways for which the target and *AKAP13* work were obtained from STRING [27]. All drugs and clinical indications were obtained from DrugBank [30] except for GSK-690693, which was obtained from ChEMBL [29].

Target	Target name	Evidence for interaction with *AKAP13*	Pathways/Complexes/Catalysts	Compound	Compound group	Clinical indication
RHOA	Transforming protein RhoA	Protein-protein interaction (grid) by Affinity Capture-Western assay [33]<br>Protein-protein interaction (hprd) by in vivo assay [34]<br>Protein-protein interactions in non-humans	Pathway: Rho GTPase Cycle<br>Catalyst: GEFs activate RhoA,B,C<br>Catalyst: GEFs activate Rho GTPase:GDP<br>Pathway: G alpha (12/13) signalling events	Guanosine-5'-Diphosphate	Experimental	-
PRKAR2A	cAMP-dependent protein kinase type II-alpha regulatory subunit	Protein-protein interaction (dip) by biochemical assay [35]<br>Protein-protein interaction (grid) by protein-peptide assay [36]<br>Protein-protein interaction (grid) by reconstituted complex assay [37]	Complex: PKAc/RII-alpha/RII-beta/AKAP13	GEM-231	Investigational	Under investigation with solid tumours
RHOB	Rho-related GTP-binding protein RhoB	Protein-protein interactions in non-humans	Pathway: Rho GTPase cycle<br>Catalysis: GEFs activate RhoA,B,C<br>Catalysis: GEFs activate Rho GTPase:GDP<br>Pathway: G alpha (12/13) signalling events	Botulinum Toxin Type A	Approved, investigational	Cervical dystonia, axillary hyperhidrosis, strabismus, blepharospasm, wrinkles, sweating
RAC1	Ras-related C3 botulinum toxin substrate 1	Protein-protein interactions in non-humans	Catalysis: p75NTR indirectly activates RAC and Cdc42 via a guanyl-nucleotide exchange factor<br>Pathway: NRAGE signals death through JNK<br>Pathway: G alpha (12/13) signalling events	Guanosine-5'-Diphosphate	Experimental	-
				Dextromethorphan	Approved	Coughing
RAC2	Ras-related C3 botulinum toxin substrate 2	Protein-protein interactions in non-humans	Pathway: Rho GTPase cycle<br>Catalysis: GEFs activate Rho GTPase:GDP<br>Pathway: G alpha (12/13) signalling events	Dextromethorphan	Approved	Coughing
CDC42	Cell division control protein 42 homolog	Protein-protein interactions in non-humans	Pathway: Rho GTPase cycle<br>Catalysis: GEFs activate Rho GTPase:GDP	Guanosine-5'-Diphosphate	Experimental	-
				Aminophosphonic Acid-Guanylate Ester	Experimental	-
INPP5B	Type II inositol 1,4,5-trisphosphate 5-phosphatase	Protein-protein interactions in non-humans	Pathway: Rho GTPase cycle	D-Myo-Inositol-1,4-Bisphosphate	Experimental	-
PRKACB	cAMP-dependent protein kinase catalytic subunit beta	Protein-protein interactions in non-humans	Complex: PKAc/RII-alpha/RII-beta/AKAP13	Phosphonothreonine	Experimental	-
				GSK-690693	Investigational	Under investigation for neoplasms
PRKACG	cAMP-dependent protein kinase catalytic subunit gamma	Protein-protein interactions in non-humans	Complex: PKAc/RII-alpha/RII-beta/AKAP13	GSK-690693	Investigational	Under investigation for neoplasms
PRKAG1	5'-AMP-activated protein kinase subunit gamma-1	-	Complex: PKAc/RII-alpha/RII-beta/AKAP13	Acetylsalicylic acid (Aspirin)	Approved, vet approved	Pain, inflammation, myocardial infarction
A2M	Alpha-2-macroglobulin	-	Pathway: Rho GTPase cycle	Bacitracin	Approved, vet approved	Pneumonia, empyema, skin and eye infections
				Becaplermin	Approved, investigational	Diabetic ulcers
				Ocriplasmin	Approved	Symptomatic vitreomacular adhesion

References

1. Raghu G, Rochwerg B, Zhang Y, et al. An official ATS/ERS/JRS/ALAT clinical practice guideline: Treatment of idiopathic pulmonary fibrosis. an update of the 2011 clinical practice guideline. *American journal of respiratory and critical care medicine*. 2015;192(2):e3-e19.

2. Travis WD, King TE, Bateman ED, et al. American thoracic society/european respiratory society international multidisciplinary consensus classification of the idiopathic interstitial pneumonias. *American journal of respiratory and critical care medicine*. 2002;165(2):277-304.

3. Maher TM. PROFILEing idiopathic pulmonary fibrosis: Rethinking biomarker discovery. *Eur Respir Rev*. 2013;22(128):148-152.

4. Sudlow C, Gallacher J, Allen N, et al. UK biobank: An open access resource for identifying the causes of a wide range of complex diseases of middle and old age. *PLoS Med*. 2015;12(3):e1001779.

5. Gauderman W, Morrison J. *QUANTO 1.1: A computer program for power and sample size calculations for genetic-epidemiology studies*. 2006.

6. Delaneau O, Marchini J, Zagury J. A linear complexity phasing method for thousands of genomes. *Nature methods*. 2012;9(2):179-181.

7. Howie BN, Donnelly P, Marchini J. A flexible and accurate genotype imputation method for the next generation of genome-wide association studies. *PLoS Genet*. 2009;5(6):e1000529.

8. 1000 Genomes Project Consortium. A global reference for human genetic variation. *Nature*. 2015;526(7571):68-74.

9. UK10K Consortium. The UK10K project identifies rare variants in health and disease. *Nature*. 2015;526(7571):82-90.

10. Howie B, Fuchsberger C, Stephens M, Marchini J, Abecasis GR. Fast and accurate genotype imputation in genome-wide association studies through pre-phasing. *Nat Genet*. 2012;44(8):955-959.

11. McCarthy S, Das S, Kretzschmar W, Durbin R, Abecasis G, Marchini J. A reference panel of 64,976 haplotypes for genotype imputation. *bioRxiv*. 2016:035170.

12. Marchini J, Howie B, Myers S, McVean G, Donnelly P. A new multipoint method for genome-wide association studies by imputation of genotypes. *Nat Genet*. 2007;39(7):906-913.

13. Ma C, Blackwell T, Boehnke M, Scott LJ. Recommended joint and Meta-Analysis strategies for Case-Control association testing of single Low-Count variants. *Genet Epidemiol*. 2013;37(6):539-550.

14. Kang H. Efficient and parallelizable association container toolbox (EPACTS) 2014 retrieved from http://Genome. sph. umich. edu/wiki. .

15. Hobbs BD, De Jong K, Lamontagne M, et al. Genetic loci associated with chronic obstructive pulmonary disease overlap with loci for lung function and pulmonary fibrosis. . 2017.

16. Noth I, Zhang Y, Ma S, et al. Genetic variants associated with idiopathic pulmonary fibrosis susceptibility and mortality: A genome-wide association study. *The Lancet Respiratory Medicine*. 2013;1(4):309-317.

17. Fingerlin TE, Murphy E, Zhang W, et al. Genome-wide association study identifies multiple susceptibility loci for pulmonary fibrosis. *Nat Genet*. 2013;45(6):613-620.

18. Fingerlin TE, Zhang W, Yang IV, et al. Genome-wide imputation study identifies novel HLA locus for pulmonary fibrosis and potential role for auto-immunity in fibrotic idiopathic interstitial pneumonia. *BMC genetics*. 2016;17(1):1.

19. Westra H, Peters MJ, Esko T, et al. Systematic identification of trans eQTLs as putative drivers of known disease associations. *Nat Genet*. 2013;45(10):1238-1243.

20. Lonsdale J, Thomas J, Salvatore M, et al. The genotype-tissue expression (GTEx) project. *Nat Genet*. 2013;45(6):580-585.

21. GTEx Consortium. Human genomics. the genotype-tissue expression (GTEx) pilot analysis: Multitissue gene regulation in humans. *Science*. 2015;348(6235):648-660.

22. Hao K, Bossé Y, Nickle DC, et al. Lung eQTLs to help reveal the molecular underpinnings of asthma. *PLoS Genet*. 2012;8(11):e1003029.

23. Lamontagne M, Couture C, Postma DS, et al. Refining susceptibility loci of chronic obstructive pulmonary disease with lung eqtls. *PLoS One*. 2013;8(7):e70220.

24. Miller S, Probert K, Billington CK, et al. GSTCD and INTS12 regulation and expression in the human lung. *PLoS One*. 2013;8(9):e74630.

25. Wakefield J. A bayesian measure of the probability of false discovery in genetic epidemiology studies. *The American Journal of Human Genetics*. 2007;81(2):208-227.

26. van de Bunt M, Cortes A, Brown MA, Morris AP, McCarthy MI, IGAS Consortium. Evaluating the performance of fine-mapping strategies at common variant GWAS loci. *PLoS Genet*. 2015;11(9):e1005535.

27. Szklarczyk D, Franceschini A, Wyder S, et al. STRING v10: Protein-protein interaction networks, integrated over the tree of life. *Nucleic Acids Res*. 2015;43(Database issue):D447-52.

28. UniProt Consortium. The universal protein resource (UniProt). *Nucleic Acids Res*. 2008;36(Database issue):D190-5.

29. Gaulton A, Bellis LJ, Bento AP, et al. ChEMBL: A large-scale bioactivity database for drug discovery. *Nucleic Acids Res*. 2012;40(Database issue):D1100-7.

30. Wishart DS, Knox C, Guo AC, et al. DrugBank: A comprehensive resource for in silico drug discovery and exploration. *Nucleic Acids Res*. 2006;34(Database issue):D668-72.

31. Mushiroda T, Wattanapokayakit S, Takahashi A, et al. A genome-wide association study identifies an association of a common variant in TERT with susceptibility to idiopathic pulmonary fibrosis. *J Med Genet*. 2008;45(10):654-656.

32. Johnson AD, Handsaker RE, Pulit SL, Nizzari MM, O'Donnell CJ, de Bakker PI. SNAP: A web-based tool for identification and annotation of proxy SNPs using HapMap. *Bioinformatics*. 2008;24(24):2938-2939.

33. Abdul Azeez KR, Knapp S, Fernandes JM, Klussmann E, Elkins JM. The crystal structure of the RhoA-AKAP-lbc DH-PH domain complex. *Biochem J*. 2014;464(2):231-239.

34. Tan Y, Wu H, Wang W, Zheng Y, Wang Z. Characterization of the interactions between the small GTPase RhoA and its guanine nucleotide exchange factors. *Anal Biochem*. 2002;310(2):156-162.

35. Hausken ZE, Coghlan VM, Hastings CA, Reimann EM, Scott JD. Type II regulatory subunit (RII) of the cAMP-dependent protein kinase interaction with A-kinase anchor proteins requires isoleucines 3 and 5. *J Biol Chem*. 1994;269(39):24245-24251.

36. Alto NM, Soderling SH, Hoshi N, et al. Bioinformatic design of A-kinase anchoring protein-in silico: A potent and selective peptide antagonist of type II protein kinase A anchoring. *Proc Natl Acad Sci U S A*. 2003;100(8):4445-4450.

37. Carr DW, Hausken ZE, Fraser ID, Stofko-Hahn RE, Scott JD. Association of the type II cAMP-dependent protein kinase with a human thyroid RII-anchoring protein. cloning and characterization of the RII-binding domain. *J Biol Chem*. 1992;267(19):13376-13382.
